# Insulin resistance prior to term age in very low birthweight infants: a prospective study

**DOI:** 10.1136/bmjpo-2023-002470

**Published:** 2024-02-10

**Authors:** Itay Zamir, Elisabeth Stoltz Sjöström, Johannes van den Berg, Estelle Naumburg, Magnus Domellöf

**Affiliations:** 1 Department of Clinical Sciences, Pediatrics, Umeå University, Umeå, Sweden; 2 Department of Food, Nutrition and Culinary Science, Umeå University, Umeå, Sweden

**Keywords:** endocrinology, neonatology

## Abstract

**Objective:**

To explore the glucose-related hormone profile of very low birthweight (VLBW) infants and assess the association between neonatal hyperglycaemia and insulin resistance during the admission period.

**Design:**

A prospective observational study—the Very Low Birth Weight Infants, Glucose and Hormonal Profiles over Time study.

**Setting:**

A tertiary neonatal intensive care unit and four neonatal units in county hospitals in Sweden.

**Patients:**

48 infants born <1500 g (VLBW) during 2016–2019.

**Outcome measures:**

Plasma concentrations of glucose-related hormones and proteins (C-peptide, insulin, ghrelin, glucagon-like peptide 1 (GLP-1), glucagon, leptin, resistin and proinsulin), insulin:C-peptide and proinsulin:insulin ratios, Homoeostatic Model Assessment 2 (HOMA2) and Quantitative Insulin Sensitivity Check (QUICKI) indices, measured on day of life (DOL) 7 and at postmenstrual age 36 weeks.

**Results:**

Lower gestational age was significantly associated with higher glucose, C-peptide, insulin, proinsulin, leptin, ghrelin, resistin and GLP-1 concentrations, increased HOMA2 index, and decreased QUICKI index and proinsulin:insulin ratio. Hyperglycaemic infants had significantly higher glucose, C-peptide, insulin, leptin and proinsulin concentrations, and lower QUICKI index, than normoglycaemic infants. Higher glucose and proinsulin concentrations and insulin:C-peptide ratio, and lower QUICKI index on DOL 7 were significantly associated with longer duration of hyperglycaemia during the admission period.

**Conclusions:**

VLBW infants seem to have a hormone profile consistent with insulin resistance. Lower gestational age and hyperglycaemia are associated with higher concentrations of insulin resistance markers.

**WHAT IS ALREADY KNOWN ON THIS TOPIC**

- Hyperglycaemia, a condition associated with many detrimental complications, is common in preterm infants during the postnatal period but its cause is debated.
- Children born preterm are more prone to develop insulin resistance during their lifetime.

**WHAT THIS STUDY ADDS**

- Very low birthweight infants seem to have laboratory signs of insulin resistance early in life, already during the admission period at the neonatal intensive care unit, with hyperglycaemic infants having higher levels of insulin resistance markers than normoglycaemic infants.

**HOW THIS STUDY MIGHT AFFECT RESEARCH, PRACTICE OR POLICY**

- The study supplies data regarding hormone levels in preterm infants and markers that might be associated with hyperglycaemia later during the admission period, which might be of use for clinicians.
- The study reports data supporting the theory that the mechanism of neonatal hyperglycaemia is related to increased insulin resistance, which might be useful for future research of possible treatment methods.

## Introduction

Hyperglycaemia is common in preterm born infants. Up to 80% of extremely preterm infants (<28 gestational weeks) and very low birthweight infants (VLBW; <1500 g) are exposed to hyperglycaemia during the first week of life, and the condition is common later during the admission period as well.^1–3^ Hyperglycaemia is also associated with a multitude of adverse outcomes, including increased mortality and impaired neurodevelopment.^3–11^ It is well known that children born preterm have increased risk of insulin resistance and metabolic syndrome later in life.^12–21^

The mechanisms responsible for hyperglycaemia in the preterm infant are not fully understood. Reduced peripheral insulin sensitivity has been suggested as a possible mechanism.^22–25^ Another possible explanation suggested is beta cell dysfunction, either due to reduced beta cell mass in the pancreas^26^ or aberrant processing of proinsulin in these cells which leads to relative insulin deficiency.^27^ A deficient incretin response due to late onset of enteral feeds,^28–30^ and insufficient control of glucose production are postulated mechanisms as well.^31 32^ Other perinatal clinical conditions, such as sepsis, might be involved in the pathophysiology of hyperglycaemia.^33^

Very few studies have reported hormone concentrations in preterm infants and more information is needed to clarify the mechanisms causing hyperglycaemia in these infants.

This study aimed at investigating the glucose-related hormone profile of VLBW infants during the admission period at the neonatal intensive care unit (NICU), including possible associations with hyperglycaemia.

## Methods

### Study population

A prospective cohort study—The Very Low Birth Weight Infants, Glucose and Hormonal Profiles over Time (LIGHT)—included VLBW infants admitted to the NICU at the tertiary university hospital in Umeå, Sweden, between 1 October 2016 and 30 November 2019. Infants were included within the first week after birth if they were transported to the NICU within 24 hours after birth, remained at the NICU for the first week of life, did not have congenital malformations or chromosomal aberrations and were registered at the four participating county hospitals.

**Figure 1:**
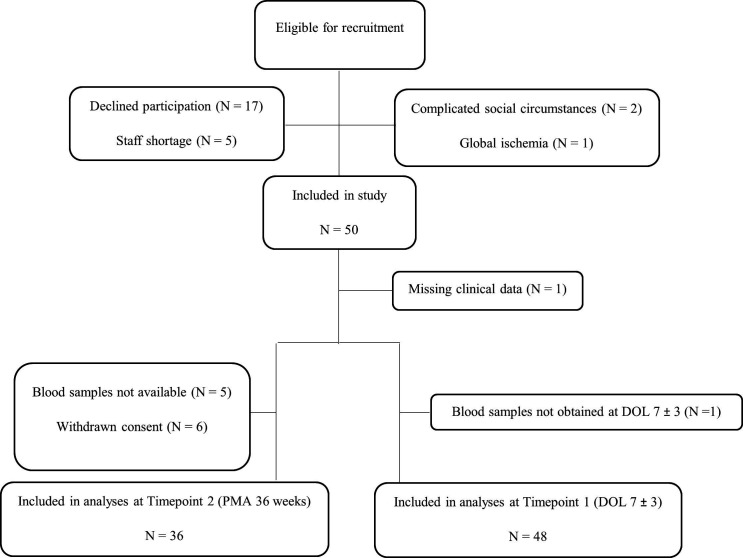
Included and excluded very low birthweight infants. DOL, day of life; PMA, postmenstrual age.

Seventy-five infants were eligible for recruitment. Of these, 67 were approached (8 were not approached due to staff shortage, complicated social circumstances and extensive brain injury in the infant) and 50 (75%) approved participation (figure 1). Two infants (4%) died before reaching postmenstrual age (PMA) 36 weeks and 6 infants (12%) left the study due to withdrawal of consent before reaching this time point.

### Study procedure

Blood samples (0.6 mL whole blood) were obtained from the infants on day of life (DOL) 7±3 days (time point 1; T1) and at PMA 36±1 weeks (time point 2; T2). The samples were obtained just before enteral feedings, together with clinically indicated blood samples. Parenteral nutrition was administered to some extent in 44 infants at T1 and in 2 infants at T2 at the time of sampling. No infant was receiving inotrope or corticosteroid treatment when the samples were obtained. All blood sampled at T1 were obtained at the NICU in Umeå while blood samples at T2 were obtained at all participating hospitals. When possible, topical anaesthetics were used prior to venipuncture. After a coagulation time of approximately 1 hour, the samples were centrifuged, and serum was separated into two 150 µL tubes. The tubes were immediately stored in a freezer at −80°C. No protease inhibitor was added.

One infant (2%) was excluded from all analyses due to missing clinical data (figure 1). One infant (2%) was excluded from analyses regarding T1 due to blood sample obtained out of given time frame. Thus, blood samples at T1 from 48 infants (96%) were included in the analyses. Samples from 41 infants (82%) were obtained at T2, of which 5 samples were not available for analysis. Thus, blood samples at T2 from 36 infants (72%) were included in the analyses. In five of them, glucose concentrations were not obtained simultaneously as blood sampling and these infants were thus excluded from analyses involving glucose concentrations.

### Glucose-related hormone profile

Serum samples were thawed and analysed for concentrations of C-peptide, insulin, ghrelin, gastric inhibitory polypeptide (GIP), glucagon-like peptide 1 (GLP-1), glucagon, leptin, resistin and visfatin using Bio-Plex Pro human diabetes multiplex immunoassay (Bio-Rad Laboratories, Hercules, California). Interassay variation was 1%–6% for all analytes except ghrelin (15%), and intraassay variation was below 15%. Proinsulin concentrations were measured using an ELISA (EMD Millipore, St. Louis, Missouri). Interassay variation was 4% and intraassay variation was 1%–9%.

Insulin:C-peptide and proinsulin:insulin ratios were calculated. Homoeostatic Model Assessment 2 (HOMA2) index was calculated using a calculator released by the Diabetes Trials Unit, University of Oxford: HOMA Calculator. The calculator is available at: http://www.dtu.ox.ac.uk/homacalculator/index.php (V.2.2.4, updated 27 November 2019). Quantitative Insulin Sensitivity Check Index (QUICKI) was calculated using the formula:

1/(log(insulinμU/mL)+log(glucosemg/dL))

### Perinatal data

Comprehensive perinatal data, including daily glucose values and neonatal morbidities, were collected prospectively from medical notes and are listed in table 1. Hyperglycaemia was defined as any single glucose concentration >10 mmol/L.^5^ Local guidelines at the NICU recommend starting insulin infusion if glucose concentration is above 13 mmol/L and decreasing glucose intake has not remedied the hyperglycaemia. No sample analysed for glucose-related hormone concentrations was taken while insulin treatment was ongoing.

**Table 1:** Cohort characteristics

Characteristic	DOL 7 (Time point 1) N=48	PMA 36 weeks (Time point 2) N=36
Perinatal variables		
Gestational age, weeks, mean (SD)	27.2 (2.5)	27.3 (2.7)
Extremely preterm (<28 weeks), N (%)	31 (65)	23 (64)
Male sex, N (%)	14 (29)	11 (31)
Multiple gestation, N (%)	6 (13)	3 (8)
Small for gestational age*, N (%)	20 (42)	16 (44)
Antenatal steroid treatment, N (%)	44 (92)	34 (94)
Amnionitis, N (%)	4 (8)	4 (11)
Pre-eclampsia, N (%)	11 (23)	8 (22)
Caesarean section, N (%)	36 (75)	27 (75)
Apgar 1 min, mean (SD)	5 (3)	5 (2)
Apgar 5 min, mean (SD)	7 (2)	8 (2)†
Apgar 10 min, mean (SD)	8 (2)	8 (1)
Anthropometric variables at birth		
Weight, grams, mean (SD)	936 (280)	925 (296)
Weight z-score, mean (SD)	−1.27 (1.52)	−1.51 (1.61)
Length, cm, mean (SD)	34.7 (3.4)	34.6 (3.7)
Length z-score, mean (SD)	−1.69 (1.91)	−1.88 (2.03)
Head circumference, cm, mean (SD)	24.4 (2.4)	24.4 (2.5)
Head circumference z-score, mean (SD)	−0.95 (0.98)	−1.05 (1.05)
Neonatal variables		
Duration (in days) of mechanical ventilation treatment during admission period, mean (SD)	8.9 (13.6)	8.7 (13.3)
Inotrope treatment, N (%)	7 (15)	4 (11)
Treatment with systemic steroids, N (%)	11 (23)	8 (22)
Insulin treatment, N (%)	9 (19)	7 (19)
Culture-verified sepsis during admission period, N (%)	5 (10)	4 (11)
Intraventricular haemorrhage		
None, N (%)	35 (73)	26 (72)
Grade 1–2, N (%)	7 (15)	6 (17)
Grades 3–4, N (%)	6 (13)	4 (11)
Necrotising enterocolitis, N (%)	1 (2)	0 (0)
Patent ductus arteriosus, N (%)	29 (60)	18 (50)†
Periventricular leukomalacia, N (%)	1 (2)	1 (3)
Retinopathy of prematurity		
None, N (%)	30 (63)	22 (61)
Stages 1–2, N (%)	9 (19)	6 (17)
Stages 3–5, N (%)	9 (19)	8 (22)
Bronchopulmonary dysplasia, N (%)	19 (40) (N=47)	16 (44)

*Defined as birth weight <10 th %-ile.

†P<0.05 when comparing infants remaining in the study and excluded infants at Time point 2.

DOL, day of life; PMA, postmenstrual age.

### Statistical analyses

The analyses were done using SPSS Statistical software (IBM, Released 2020. IBM SPSS Statistics for Windows, V.27.0., IBM). Student’s t-test and Mann-Whitney U-test with calculations of SD and IQRs were used as appropriate. Generalised linear mixed models with a linear link function were used to assess associations between gestational age at birth and concentrations of glucose-related hormones at T1 and T2. Linear regression was used to evaluate the associations between concentrations of glucose-related hormones at T1 and number of days with hyperglycaemia afterwards (rest of the admission period). The significance level was set to p<0.05.

### Patient and public involvement

Patients or the public were not involved in the design, or conduct, or reporting, or dissemination plans of our research.

## Results

Cohort characteristics and anthropometric data are presented in table 1. Statistically significant differences between infants included at T1 (DOL 7±3 days) and T2 (PMA 36±1 weeks) included higher Apgar scores at 5 min and lower prevalence of patent ductus arteriosus in the infants analysed at T2.

### Concentrations of glucose-related hormones

Concentrations of glucose-related hormones at T1 and T2 in the entire cohort are presented in table 2. Quantifiable concentrations of visfatin were found only in one infant (2%) at T1 and in none (0%) at T2 (lower limit of quantification (LLOQ) 35.6 pg/mL) and are, therefore, not presented. Quantifiable concentrations of GIP were found only in 1 infant (2%) at T1 and in 6 infants (17%) at T2 (LLOQ 8.5 pmol/L), which was deemed to be too few samples to proceed with statistical analyses.

**Table 2:** Concentrations of glucose-related hormones in very low birthweight infants at day of life 7±3 (time point 1) and at postmenstrual age 36±1 weeks (time point 2)

	Time point 1 N=48	Time point 2 N=36
C-peptide, pmol/L, median (IQR)	440.5 (253.6–618.8)	274.7 (156.9–537.6)
Insulin, pmol/L, median (IQR)	183.8 (108.7–288.4)	80.3 (39.6–202.3)
Insulin:C-peptide, median (IQR)	0.42 (0.34–0.55)	0.35 (0.25–0.47)
Glucose, mmol/L, median (IQR)	6.5 (5.7–8.5)	5.7 (4.9–6.7)
HOMA2, median (IQR)	3.6 (2.1–5.8)	1.6 (0.8–3.8)
QUICKI, median (IQR)	0.28 (0.27–0.30)	0.32 (0.29–0.36)
Proinsulin, pmol/L, median (IQR)	42.6 (31.5–61.8)	13.4 (10.0–21.0)
Proinsulin:insulin, median (IQR)	0.27 (0.19–0.34)	0.14 (0.09–0.30)
Leptin, µg/L, median (IQR)*	0.11 (0.05–0.25)	0.55 (0.33–1.18)
Ghrelin, pmol/L, median (IQR)	1326.3 (1029.0–1694.8)	940.2 (826.9–1110.2)
GLP1, pmol/L, median (IQR)	318.3 (230.7–428.9)	261.7 (194.6–366.1)
Resistin, ng/mL, median (IQR)	29.9 (24.8–36.2)	17.9 (15.1–25.3)
Glucagon, ng/L, mean (SD)	1175.1 (264.6)	950.2 (254.0)

*Quantifiable concentrations of leptin were found in 12 infants (25%) at time point 1 and in 33 infants (92%) at time point 2 (lower limit of quantification 0.01 µg/L).

GLP-1, glucagon-like peptide; HOMA2, Homoeostatic Model Assessment 2; QUICKI, Quantitative Insulin Sensitivity Check Index.

**Figure 2:**
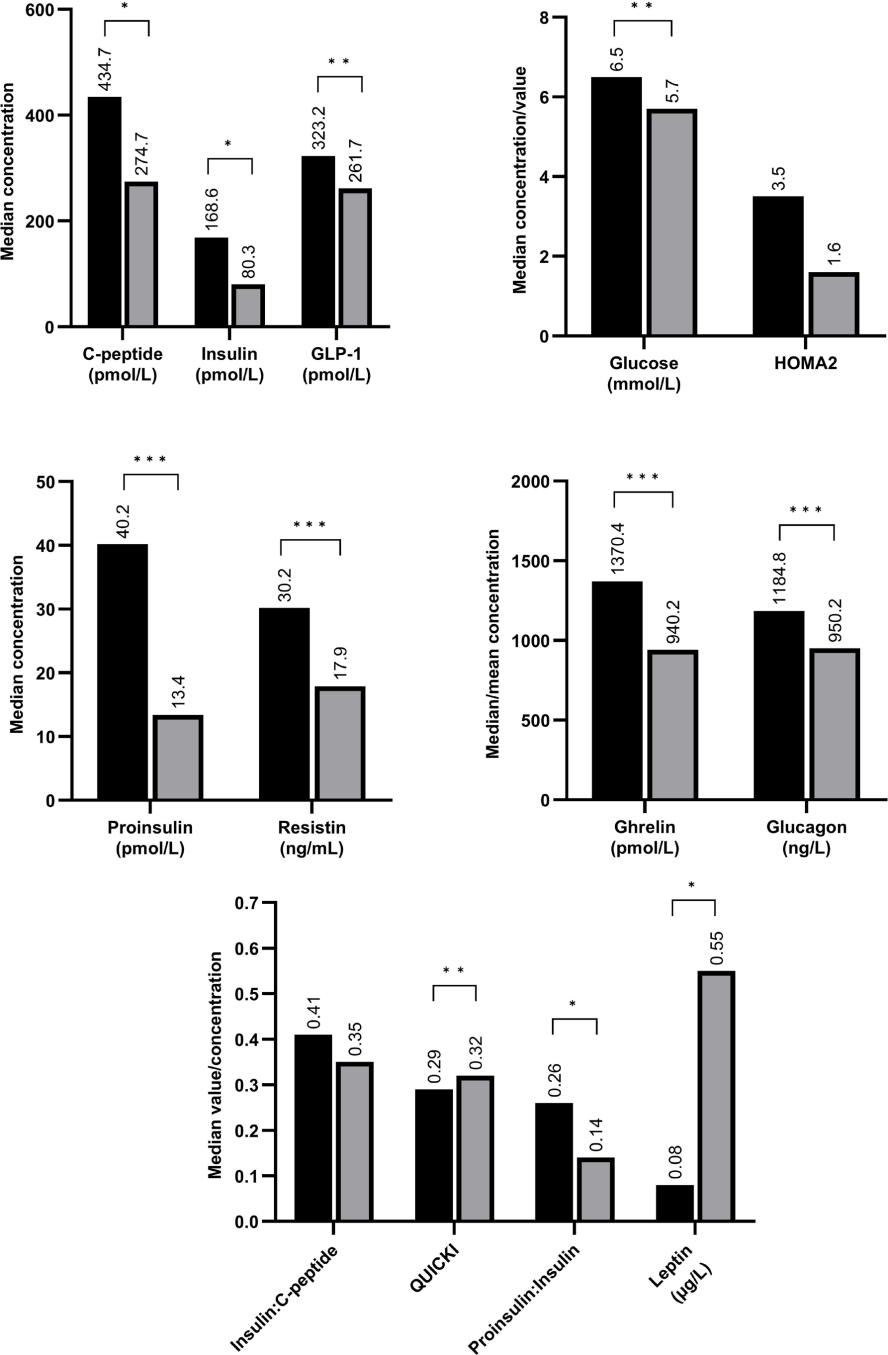
Concentrations of glucose-related hormones in very low birthweight infants on day of life 7±3 (time point 1; black) compared with concentrations at postmenstrual age 36±1 weeks (time point 2; grey). *p<0.05; **p<0.01; ***p<0.001. GLP-1, glucagon-like peptide 1; HOMA2, Homoeostatic Model Assessment 2; QUICKI, Quantitative Insulin Sensitivity Check Index.

To evaluate change in glucose-related hormone concentrations over time, we compared concentrations between infants for whom samples were available at both time points (N=36; N=7 for leptin). The results are presented in figure 2. Concentrations of C-peptide, insulin, glucose, proinsulin, ghrelin, GLP-1, resistin and glucagon as well as proinsulin:insulin ratio decreased significantly between T1 and T2, while leptin concentrations and QUICKI value increased significantly.

### Gestational age at birth and glucose-related hormone concentrations

Associations between gestational age at birth and glucose-related hormone concentrations at T1 and T2 are presented in online supplemental file 1. At T1, higher gestational age at birth was significantly associated with lower concentrations of glucose, ghrelin, and resistin, and higher concentrations of GLP-1. At T2, higher gestational age at birth was significantly associated with decreased concentrations of insulin and leptin, and increased proinsulin:insulin ratio. At both time points, higher gestational age at birth was significantly associated with lower concentrations of C-peptide and proinsulin, decreased HOMA2 index and increased QUICKI value.

10.1136/bmjpo-2023-002470.supp1 Supplementary data

### Glucose-related hormone concentrations and hyperglycaemia

**Figure 3:**
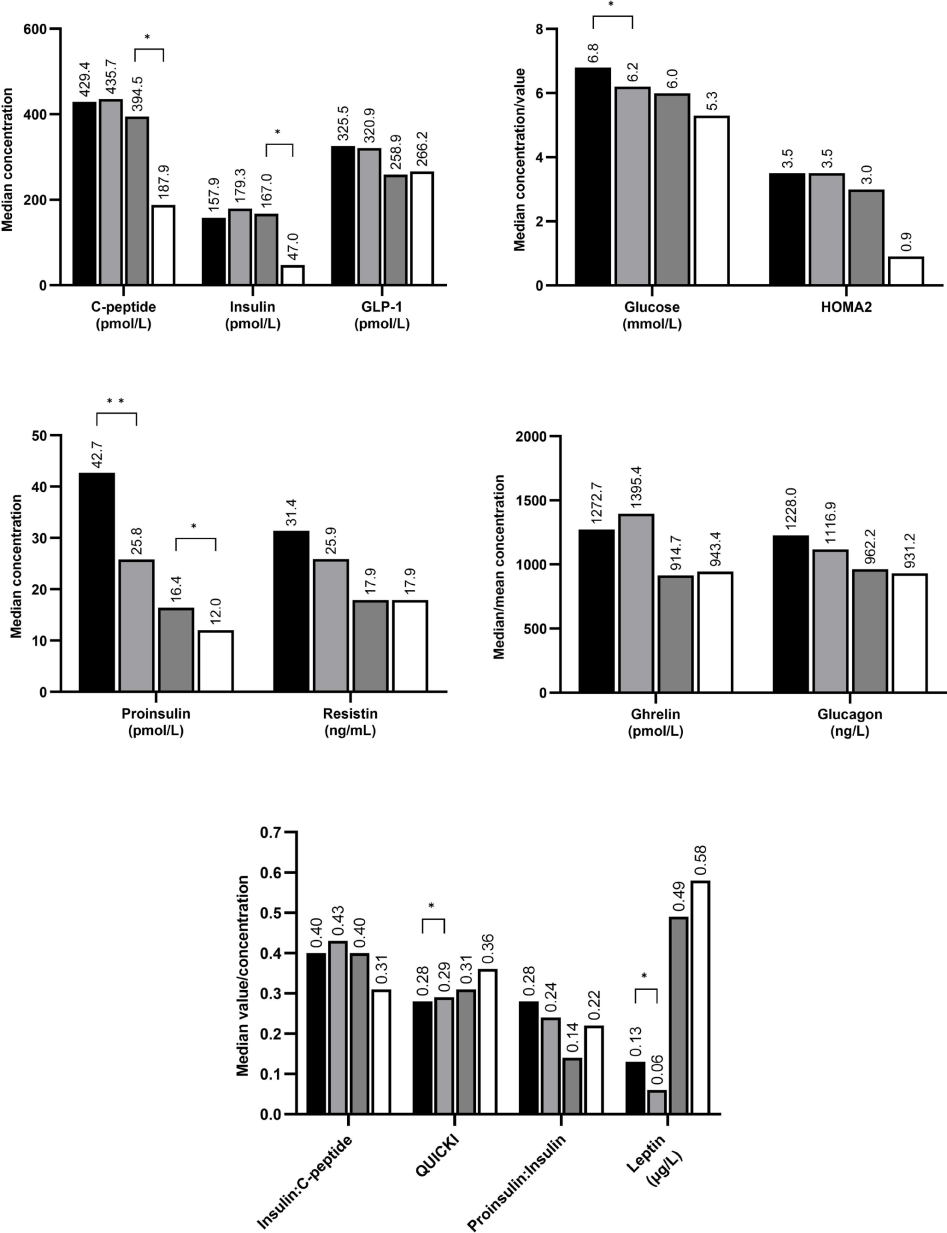
Concentrations of glucose-related hormones in hyperglycaemic and normoglycaemic very low birthweight infants sampled on day of life 7±3 (time point 1; black and light grey, respectively) and at postmenstrual age 36±1 weeks (time point 2; dark grey and white, respectively). *p<0.05; **p<0.01. GLP-1, glucagon-like peptide 1; HOMA2, Homoeostatic Model Assessment 2; QUICKI, Quantitative Insulin Sensitivity Check Index.

At T1, hyperglycaemic infants (N=30) had significantly higher glucose and leptin concentrations than normoglycaemic infants (N=18), as well as significantly lower QUICKI value (figure 3). At T2, hyperglycaemic infants (N=22) had significantly higher C-peptide and insulin concentrations than normoglycaemic infants (N=14). At both time points, hyperglycaemic infants had significantly higher proinsulin concentrations than normoglycaemic infants.

### Associations between glucose-related hormone concentrations at T1 and duration of subsequent hyperglycaemia

Higher glucose and proinsulin concentrations and insulin:C-peptide ratio at T1, as well as lower QUICKI value, were significantly associated with more days with hyperglycaemia >10 mmol/L during the rest of the admission period (after T1; online supplemental file 2).

10.1136/bmjpo-2023-002470.supp2 Supplementary data

## Discussion

This prospective study reports concentrations of a broad range of glucose-related hormones in VLBW infants at two time points: DOL 7±3 days and PMA 36±1 weeks. Insulin resistance markers decreased while insulin sensitivity markers increased between the two time points. Prematurity was associated with higher concentrations of insulin resistance markers. Compared with normoglycaemic infants, hyperglycaemic infants had higher concentrations of a number of insulin resistance markers. Higher glucose and proinsulin concentrations and insulin:C-peptide ratio and lower QUICKI value at DOL 7 were associated with longer periods of hyperglycaemia during the remaining admission period.

Glucose-related hormone concentrations reported in this study were higher than those reported in previous literature. For example, Salis *et al* reported lower insulin, glucose, C-peptide and glucose concentrations, and higher GLP-1 concentrations and insulin:C-peptide ratio.^28^ However, substantial methodical differences (method of laboratory test, timing of blood sampling, population selection) render a comparison of such results difficult. No previous study has reported glucose-related hormone concentrations at precisely the same time points as the current study. Furthermore, the LIGHT study included the largest proportion of extremely preterm infants compared with other published literature.

Concentrations of C-peptide, insulin, glucose, proinsulin, ghrelin, resistin and glucagon, as well as proinsulin:insulin ratio, have significantly decreased from DOL 7 to PMA 36 weeks, while leptin concentrations and QUICKI value significantly increased. This can be interpreted as an improvement in insulin resistance status, with a decrease in insulin resistance-promoting hormones and an increase in insulin sensitivity markers as the infant matures and the nutritional status improves. Unexpectedly, concentrations of GLP-1, an incretin hormone which is secreted in response to increased enteral nutrition and decreases insulin resistance, decreased with time. This might be due to the overall trend of decrease in insulin resistance with time which in turn would decrease the need in a GLP-1 response. These findings should be confirmed in future larger studies.

In our study, lower gestational age at birth was significantly associated with higher concentrations of glucose, ghrelin and resistin and lower concentrations of GLP-1 on DOL 7; with higher concentrations of insulin and leptin and decreased proinsulin:insulin ratio at PMA 36 weeks; and with higher concentrations of C-peptide and proinsulin, increased HOMA2 index and decreased QUICKI index at both time points. Overall, these results suggest an association between lower gestational age at birth and increased insulin resistance, both early in life and when nearing term age. Salis *et al* have previously shown in a smaller study population that lower gestational age at birth was associated with higher insulin and glucose concentrations.^25 28^

Compared with normoglycaemic infants, hyperglycaemic infants had significantly higher concentrations of glucose and leptin and lower QUICKI value on DOL 7; higher concentrations of C-peptide and insulin at PMA 36 weeks; and higher concentrations of proinsulin at both time points. These results are supported by findings from two smaller previous studies.^25 27^ This suggests that insulin resistance might be the mechanism responsible for hyperglycaemia. Of note, there was no significant difference in proinsulin:insulin ratio between the groups, which does not support the theory of aberrant proinsulin processing as a cause of hyperglycaemia.^27^ Another important finding is the relatively high HOMA2 scores noted in the different infant groups while mean glucose concentrations were not in the hyperglycaemic range. This might suggest that VLBW infants have a subclinical insulin resistance even in the absence of overt hyperglycaemia.

The LIGHT-study also presents evidence for an association between glucose-related hormone concentrations and later hyperglycaemia. Higher concentrations of glucose and proinsulin measured on DOL 7, as well as higher insulin:C-peptide ratio and lower QUICKI value (based on glucose and insulin concentrations) at the same time point, were significantly associated with higher number of days with hyperglycaemia after this time point. This suggests that measuring these parameters at DOL 7 might help identify those infants that are most in need of close monitoring of glucose concentrations. Further studies are needed to explore the feasibility of using these markers to identify infants who have higher risk for prolonged hyperglycaemia.

Only 25% of infants had quantifiable leptin concentrations on DOL 7 which increased to 92% of infants at PMA 36 weeks. Two previous studies in preterm infants have reported similar leptin concentrations in the first days of life.^34 35^ Siahanidou *et al* on the other hand reported somewhat higher leptin concentrations at discharge (mean DOL 41) in preterm infants.^36^ The LIGHT study included a higher proportion of extremely preterm infants which could explain the difference in the results, supporting in part the finding that more premature infants have increased insulin resistance and reflecting the poor nutritional status of these infants shortly after birth and its improvement during the admission period.

The LIGHT study was a prospective study focused on glucose metabolism in VLBW infants. The many different hormonal pathways that affect glucose metabolism are intricately intertwined and this study is the first to report the results of a broad glucose-related hormone panel in this patient population. Furthermore, results are reported at two different time points, providing unique information regarding trends over time. To the best of our knowledge, it is the largest study to date to compare glucose-related hormone concentrations between hyperglycaemic and normoglycaemic infants. It is also the first study to report glucose-related hormone concentrations in preterm infants measured at the same predefined PMA nearing term age.

Although the largest to-date of its kind, the number of infants included in this study was relatively small, and larger studies are needed to confirm the findings. As the NICU at Umeå university hospital is one of six designated NICUs in Sweden providing care for extremely preterm infants, the LIGHT study included a majority of extremely preterm infants (as opposed to VLBW). It should be noted that HOMA2 and QUICKI indices, as well as the interpretation of glucose-related hormone concentrations in relation to one another in general, should usually be done using blood samples obtained in a fasting state. It would be unethical to implement this method in preterm infants and we have, therefore, used blood samples obtained just before enteral feedings which reflects the actual clinical situation in the NICU. Most of the infants received parenteral nutrition to some degree at T1, and two infants received parenteral nutrition at T2. This might have affected the concentrations measured to some extent. As mentioned earlier, the different hormonal pathways affecting glucose metabolism are complicated and difficult to isolate from one another. The results should, therefore, be interpreted with caution since it is debated whether insulin resistance markers (even in the fasting state) fully reflect the insulin resistance status in lean children.

### Conclusion

VLBW infants seem to have a glucose-related hormone profile consistent with insulin resistance, both early in life and when nearing term age. Hyperglycaemic preterm infants have higher concentrations of insulin resistance markers than normoglycaemic infants. Lower gestational age is associated with higher concentrations of insulin resistance markers and insulin resistance decreases when nearing term age. Glucose and proinsulin concentrations, insulin:C-peptide ratio and QUICKI value might be useful markers to identify infants at risk for recurring hyperglycaemia.

## Supplementary Material

Reviewer comments

Author's manuscript

## Data Availability

Data are available on reasonable request. Deidentified participant data are available after contact with the corresponding author (see above for contact details). Reuse of data is only permitted after discussion with the corresponding author.
