# Supplementary material for: Insulin resistance prior to term age in very low birthweight infants: a prospective study
Source: BMJ Paediatr Open. 2024 Feb 10;8(1):e002470. doi: 10.1136/bmjpo-2023-002470 (PMC10862284; doi:10.1136/bmjpo-2023-002470)
Supplement: Supplementary data [file bmjpo-2023-002470supp001.pdf]

Supplementary file 1. Associations between gestational age at birth and glucose-related hormone concentrations on day of life 7±3 (Timepoint 1) and at postmenstrual age 36±1 weeks (Timepoint 2) in very low birth weight infants.

	**Timepoint 1**			**Timepoint 2**		
	Coefficient	95% CI	P value	Coefficient	95% CI	P value
C-peptide	-39.7	-72.3 to -7.2	**0.02**	-51.8	-81.8 to -21.9	**0.001**
Insulin	-16.6	-34.3 to 1.2	0.07	-29.9	-49.0 to -10.7	**0.003**
Insulin: C-peptide	-0.01	-0.03 to 0.01	0.43	-0.02	-0.04 to 0.01	0.13
Glucose	-0.4	-0.7 to -0.2	**0.003**	-0.2	-0.4 to 0.01	0.07
HOMA2	-0.4	-0.7 to -0.03	**0.03**	-0.7	-1.1 to -0.3	**0.001**
QUICKI	0.006	0.002 to 0.01	**0.002**	0.012	0.005 to 0.018	**0.001**
Proinsulin	-6.1	-8.4 to -3.8	**< 0.001**	-1.4	-2.5 to -0.3	**0.01**
Proinsulin: Insulin	-0.01	-0.05 to 0.02	0.44	0.02	0.00 to 0.04	**0.048**
Leptin*	-0.06	-0.29 to 0.16	0.55	-0.15	-0.2 to -0.07	**0.001**
Ghrelin	-79.0	-141.2 to -16.8	**0.01**	-1.8	-62.1 to 58.4	0.95
GLP-1	20.1	0.1 to 40.1	**0.049**	1.8	-14.5 to 18.2	0.82
Resistin	-1.6	-2.6 to -0.6	**0.002**	-0.1	-1.0 to 0.9	0.88

Glucagon	-4.4	-37.7 to 29.0	0.79	12.3	-20.8 to 45.4	0.45

* Quantifiable concentrations of leptin were found in 12 infants (25%) at Timepoint 1 and in 33 infants (92%) at Timepoint 2 (LLOQ 0.01 mcg/L). HOMA2 – homeostatic model assessment 2; QUICKI – quantitative insulin sensitivity check index; GLP-1 – glucagon-like peptide.
