# Supplementary material for: Insulin resistance prior to term age in very low birthweight infants: a prospective study
Source: BMJ Paediatr Open. 2024 Feb 10;8(1):e002470. doi: 10.1136/bmjpo-2023-002470 (PMC10862284; doi:10.1136/bmjpo-2023-002470)
Supplement: Supplementary data [file bmjpo-2023-002470supp002.pdf]

Supplementary file 2. Associations between glucose-related hormone concentrations on day of life 7±3 (Timepoint 1) and duration of subsequent hyperglycemia in very low birth weight infants.

**Glucose-related parameter (increase in value measured at Timepoint 1)**	**N days with hyperglycemia after Timepoint 1**			
	B	95% CI	P value	R^2
C-peptide (+100 pmol/L)	0.1	-0.5 to 0.7	0.68	0.004
Insulin (+100 pmol/L)	0.7	-0.3 to 1.8	0.18	0.04
Insulin:C-peptide (+0.1)	1.48	0.69 to 2.28	**< 0.001**	0.24
Glucose (+1 mmol/L)	1.26	0.71 to 1.81	**< 0.001**	0.32
HOMA (+1)	0.48	-0.06 to 1.02	0.08	0.07
QUICKI (+0.01)	-0.48	-0.91 to -0.05	**0.03**	0.08
Proinsulin (+10 pmol/L)	0.7	0.03 to 1.4	**0.04**	0.09
Proinsulin:insulin (+0.1)	0.03	-0.56 to 0.62	0.92	0.0
Leptin (+0.1 mcg/L)	-0.06	-0.64 to 0.51	0.82	0.001
Ghrelin (+100 pmol/L)	0.2	-0.04 to 0.5	0.09	0.06
GLP-1 (+1 pmol/L)	0.0	-0.01 to 0.01	0.95	0.0
Resistin (+1 ng/mL)	0.11	-0.08 to 0.29	0.25	0.03
Glucagon (+100 ng/L)	0.1	-0.5 to 0.7	0.84	0.001

HOMA2 – homeostatic model assessment 2; QUICKI – quantitative insulin sensitivity check index; GLP-1 – glucagon-like peptide.
