# Supplementary material for: Insulin resistance prior to term age in very low birthweight infants: a prospective study
Source: BMJ Paediatr Open. 2024 Feb 10;8(1):e002470. doi: 10.1136/bmjpo-2023-002470 (PMC10862284; doi:10.1136/bmjpo-2023-002470)
Supplement: Author's manuscript [file bmjpo-2023-002470.draft_revisions.pdf]

BMJ Paediatrics Open

BMJ Paediatrics Open is committed to open peer review. As part of this commitment we make the peer review history of every article we publish publicly available.

When an article is published we post the peer reviewers' comments and the authors' responses online. We also post the versions of the paper that were used during peer review. These are the versions that the peer review comments apply to.

The versions of the paper that follow are the versions that were submitted during the peer review process. They are not the versions of record or the final published versions. They should not be cited or distributed as the published version of this manuscript.

BMJ Paediatrics Open is an open access journal and the full, final, typeset and author-corrected version of record of the manuscript is available on our site with no access controls, subscription charges or pay-per-view fees (http://bmjpaedsopen.bmj.com).

If you have any questions on BMJ Paediatrics Open's open peer review process please email info.bmjpo@bmj.com

BMJ Paediatrics Open

Insulin resistance prior to term age in very low birth infants: a prospective study

Journal:	*BMJ Paediatrics Open*
Manuscript ID	bmjpo-2023-002470
Article Type:	Original research
Date Submitted by the Author:	20-Dec-2023

Complete List of Authors:	Zamir, Itay; Umeå University, Department of Clinical Sciences, Pediatrics<br>Stoltz Sjöström, Elisabeth; Umeå University, Department of Food, Nutrition and Culinary Science<br>van Den Berg, Johannes; Umeå University, Department of Clinical Sciences, Pediatrics<br>Naumburg, Estelle; Umeå Universitet, Department of Clinical Sciences, Pediatrics<br>Domellof, Magnus; Umeå Universitet, Department of Clinical Sciences, Pediatrics

Keywords:	Endocrinology, Neonatology

SCHOLARONE™
Manuscripts

I, the Submitting Author has the right to grant and does grant on behalf of all authors of the Work (as defined in the below author licence), an exclusive licence and/or a non-exclusive licence for contributions from authors who are: i) UK Crown employees; ii) where BMJ has agreed a CC-BY licence shall apply, and/or iii) in accordance with the terms applicable for US Federal Government officers or employees acting as part of their official duties; on a worldwide, perpetual, irrevocable, royalty-free basis to BMJ Publishing Group Ltd ("BMJ") its licensees and where the relevant Journal is co-owned by BMJ to the co-owners of the Journal, to publish the Work in this journal and any other BMJ products and to exploit all rights, as set out in our licence.

The Submitting Author accepts and understands that any supply made under these terms is made by BMJ to the Submitting Author unless you are acting as an employee on behalf of your employer or a postgraduate student of an affiliated institution which is paying any applicable article publishing charge ("APC") for Open Access articles. Where the Submitting Author wishes to make the Work available on an Open Access basis (and intends to pay the relevant APC), the terms of reuse of such Open Access shall be governed by a Creative Commons licence – details of these licences and which Creative Commons licence will apply to this Work are set out in our licence referred to above.

Other than as permitted in any relevant BMJ Author's Self Archiving Policies, I confirm this Work has not been accepted for publication elsewhere, is not being considered for publication elsewhere and does not duplicate material already published. I confirm all authors consent to publication of this Work and authorise the granting of this licence.

Confidential: For Review Only

Insulin resistance prior to term age in very low birth infants: a prospective study

Itay Zamir, MD, PhD1, Elisabeth Stoltz Sjöström, RD, PhD2, Johannes van Den Berg, RN, PhD1, Estelle Naumburg, MD, PhD1, and Magnus Domellöf, MD, PhD1.

Affiliations:
1 Department of Clinical Sciences, Pediatrics, Umeå University, Umeå, Sweden.
2 Department of Food, Nutrition and Culinary Science, Umeå University, Umeå, Sweden.

Address correspondence to: Itay Zamir, Department of Clinical Sciences, Pediatrics, Umeå University, SE-90187 Umeå, Sweden, itay.zamir@umu.se, Orcid-id: 0000-0001-9086-7991

Potential conflicts of interest: None declared.

Abbreviations:
DOL – day of life
GIP – gastric inhibitory polypeptide
GLP-1 – glucagon-like peptide 1
HOMA2 – homeostatic model assessment 2
LIGHT – very low birth weight infants, glucose and hormonal profiles over time
LLOQ – lower limit of quantification
NICU – neonatal intensive care unit
PMA – postmenstrual age
QUICKI – Quantitative insulin sensitivity check index
VLBW – very low birth weight

Keywords: very low birth weight; hyperglycemia; insulin resistance; insulin

Contributors' Statement: Dr Zamir conceptualized and designed the study, collected data as well as supervised data collection, carried out the statistical analyses, drafted the initial manuscript, and reviewed and revised the manuscript. Dr Stoltz Sjöström conceptualized and designed the study, and reviewed and revised the manuscript. Dr van Den Berg acquired blood samples, and reviewed and revised the manuscript. Dr Naumburg supervised data collection and reviewed and revised the manuscript. Prof Domellöf conceptualized and designed the study, supervised data collection, and reviewed and revised the manuscript. Dr Yonas Berhane supervised data collection in Sunderbyn county hospital. Carina Lagerquist performed laboratory analyses. Ulrica Hedman coordinated study procedures at Östersund county hospital. Ida Lönnstedt and Johanna Karlsson contributed to data collection.

All authors approved the final manuscript as submitted and agree to be accountable for all aspects of the work.

Confidential: For Review Only

Abstract

Objective: To explore the glucose-related hormone profile of very low birth weight (VLBW) infants and assess the association between neonatal hyperglycemia and insulin resistance during the admission period.

Design: A prospective observational study – the very low birth weight infants, glucose and hormonal profiles over time (LIGHT) study.

Setting: A tertiary neonatal intensive care unit (NICU) and four neonatal units in county hospitals in Sweden.

Patients: 48 infants born <1500 grams (VLBW) during 2016-2019.

Outcome measures: Plasma concentrations of glucose-related hormones and proteins (C-peptide, insulin, ghrelin, glucagon-like peptide 1 (GLP-1), glucagon, leptin, resistin, and proinsulin), insulin:C-peptide and proinsulin:insulin ratios, Homeostatic Model Assessment (HOMA2) and Quantitative Insulin Sensitivity Check (QUICKI) indeces, measured on day of life (DOL) 7 and at postmenstrual age 36 weeks.

Results: Lower gestational age was significantly associated with higher glucose, C-peptide, insulin, proinsulin, leptin, ghrelin, resistin and GLP-1 concentrations, increased HOMA2 index, and decreased QUICKI index and proinsulin:insulin ratio. Hyperglycemic infants had significantly higher glucose, C-peptide, insulin, leptin and proinsulin concentrations, and lower QUICKI index, than normoglycemic infants. Higher glucose and proinsulin concentrations and insulin:C-peptide ratio, and lower QUICKI index on DOL 7 were significantly associated with longer duration of hyperglycemia during the admission period.

Confidential: For Review Only

Conclusions: VLBW infants seem to have a hormone profile consistent with insulin resistance. Lower gestational age and hyperglycemia are associated with higher concentrations of insulin resistance markers.

What is already known on this topic:

- Hyperglycemia, a condition associated with many detrimental complications, is common in preterm infants during the postnatal period but its cause is debated.
- Children born preterm are more prone to develop insulin resistance during their lifetime.

What this study adds:

- VLBW infants seem to have laboratory signs of insulin resistance early in life, already during the admission period at the NICU, with hyperglycemic infants having higher levels of insulin resistance markers than normoglycemic infants.

How this study might affect research, practice or policy:

- The study supplies data regarding hormone levels in preterm infants and markers that might be associated with hyperglycemia later during the admission period, which might be of use for clinicians.
- The study reports data supporting the theory that the mechanism of neonatal hyperglycemia is related to increased insulin resistance, which might be useful for future research of possible treatment methods.

Confidential: For Review Only

Introduction

Hyperglycemia is common in preterm born infants. Up to 80% of extremely preterm infants (< 28 gestational weeks) and very low birth weight infants (VLBW; < 1500 g) are exposed to hyperglycemia during the first week of life, and the condition is common later during the admission period as well(1-3). Hyperglycemia is also associated with a multitude of adverse outcomes, including increased mortality and impaired neurodevelopment(3-11). It is well known that children born preterm have increased risk of insulin resistance and metabolic syndrome later in life(12-21).

The mechanisms responsible for hyperglycemia in the preterm infant are not fully understood. Reduced peripheral insulin sensitivity has been suggested as a possible mechanism(22-25). Another possible explanation suggested is beta cell dysfunction, either due to reduced beta cell mass in the pancreas(26) or aberrant processing of proinsulin in these cells which leads to relative insulin deficiency(27). A deficient incretin response due to late onset of enteral feeds(28-30), and insufficient control of glucose production are postulated mechanisms as well(31, 32). Other perinatal clinical conditions, such as sepsis, might be involved in the pathophysiology of hyperglycemia(33).

Very few studies have reported hormone concentrations in preterm infants and more information is needed to clarify the mechanisms causing hyperglycemia in these infants.

This study aimed at investigating the glucose-related hormone profile of VLBW infants during the admission period at the neonatal intensive care unit (NICU), including possible associations with hyperglycemia.

Confidential: For Review Only

Methods

Study population

A prospective cohort study - The Very Low Birth Weight Infants, Glucose and Hormonal Profiles over Time (LIGHT) - included VLBW infants admitted to the NICU at the tertiary university hospital in Umeå, Sweden, between October 1, 2016, and November 30, 2019. Infants were included within the first week after birth if they were transported to the NICU within 24 hours after birth, remained at the NICU for the first week of life, did not have congenital malformations or chromosomal aberrations, and were registered at the four participating county hospitals.

Seventy-five infants were eligible for recruitment. Of these, 67 were approached (8 were not approached due to staff shortage, complicated social circumstances, and extensive brain injury in the infant) and 50 (75%) approved participation (Figure 1). Two infants (4%) died before reaching postmenstrual age (PMA) 36 weeks and six infants (12%) left the study due to withdrawal of consent before reaching this timepoint.

Study procedure

Blood samples (0.6 mL whole blood) were obtained from the infants on day of life (DOL) 7±3 days (Timepoint 1; T1) and at PMA 36±1 weeks (Timepoint 2; T2). The samples were obtained just before enteral feedings, together with clinically indicated blood samples. Parenteral nutrition was administered to some extent in 44 infants at T1 and in 2 infants at T2 at the time of sampling. No infant was receiving inotrope or corticosteroid treatment when the samples were obtained. All blood sampled at T1 were obtained at the NICU in Umeå while blood samples at T2 were obtained at all participating hospitals. When possible, topical anesthetics were used prior to venipuncture. After a coagulation time of approximately one

https://mc.manuscriptcentral.com/bmjpo

hour, the samples were centrifuged, and serum was separated into two 150 µL tubes. The tubes were immediately stored in a freezer at -80° C. No protease inhibitor was added.

One infant (2%) was excluded from all analyses due to missing clinical data (Figure 1). One infant (2%) was excluded from analyses regarding T1 due to blood sample obtained out of given timeframe. Thus, blood samples at T1 from 48 infants (96%) were included in the analyses. Samples from 41 infants (82%) were obtained at T2, of which 5 samples were not available for analysis. Thus, blood samples at T2 from 36 infants (72%) were included in the analyses. In five of them, glucose concentrations were not obtained simultaneously as blood sampling and these infants were thus excluded from analyses involving glucose concentrations.

Glucose-related hormone profile

Serum samples were thawed and analyzed for concentrations of C-peptide, insulin, ghrelin, gastric inhibitory polypeptide (GIP), glucagon-like peptide 1 (GLP-1), glucagon, leptin, resistin and visfatin using Bio-Plex® Pro human diabetes multiplex immunoassay (Bio-Rad Laboratories, Inc. Hercules, California). Interassay variation was 1-6% for all analytes except ghrelin (15%), and intraassay variation was below 15%. Proinsulin concentrations were measured using an enzyme-linked immunosorbent assay (EMD Millipore Corp. St. Louis, Missouri). Interassay variation was 4% and intraassay variation was 1-9%.

Insulin:C-peptide and proinsulin:insulin ratios were calculated. Homeostatic Model assessment 2 (HOMA2) index was calculated using a calculator released by the Diabetes Trials Unit, University of Oxford: HOMA Calculator. The calculator is available at: http://www.dtu.ox.ac.uk/homacalculator/index.php (version 2.2.4, updated November 27, 2019). Quantitative insulin sensitivity check index (QUICKI) was calculated using the formula:

Confidential: For Review Only

$$1/(\log(insulin\ \mu U/mL) + \log(glucose\ mg/dL))$$

Perinatal data

Comprehensive perinatal data, including daily glucose values and neonatal morbidities, were collected prospectively from medical notes and are listed in Table 1. Hyperglycemia was defined as any single glucose concentration >10 mmol/L(5). Local guidelines at the NICU recommend starting insulin infusion if glucose concentration is above 13 mmol/L and decreasing glucose intake has not remedied the hyperglycemia. No sample analyzed for glucose-related hormone concentrations was taken while insulin treatment was ongoing.

Statistical analyses

The analyses were done using SPSS Statistical software (IBM corp. Released 2020. IBM SPSS Statistics for Windows, Version 27.0. Armonk, NY: IBM Corp.). Student t-test and Mann-Whitney U-test with calculations of standard deviations and interquartile ranges were used as appropriate. Generalized linear mixed models with a linear link function were used to assess associations between gestational age at birth and concentrations of glucose-related hormones at T1 and T2. Linear regression was used to evaluate the associations between concentrations of glucose-related hormones at T1 and number of days with hyperglycemia afterwards (rest of the admission period). The significance level was set to $P<0.05$.

Ethical approval

The study was approved by the regional ethical committee in Umeå, Sweden (Dnr 226-31M/2016), and written informed consent was obtained from all legal guardians.

Patient and public involvement

Confidential: For Review Only

Patients or the public were not involved in the design, or conduct, or reporting, or dissemination plans of our research.

Confidential: For Review Only

Results

Cohort characteristics and anthropometric data are presented in Table 1. Statistically significant differences between infants included at T1 (DOL 7±3 days) and T2 (PMA 36±1 weeks) included higher Apgar scores at 5 min and lower prevalence of patent ductus arteriosus in the infants analyzed at T2.

Concentrations of glucose-related hormones

Concentrations of glucose-related hormones at T1 and T2 in the entire cohort are presented in Table 2. Quantifiable concentrations of visfatin were found only in one infant (2%) at T1 and in none (0%) at T2 (Lower limit of quantification (LLOQ) 35.6 pg/mL) and are thereforbe not presented. Quantifiable concentrations of GIP were found only in one infant (2%) at T1 and in six infants (17%) at T2 (LLOQ 8.5 pmol/L), which was deemed to be too few samples to proceed with statical analyses.

To evaluate change in glucose-related hormone concentrations over time, we compared concentrations between infants for whom samples were available at both timepoints (N=36; N=7 for leptin). The results are presented in Figure 2. Concentrations of C-peptide, insulin, glucose, proinsulin, ghrelin, GLP-1, resistin and glucagon as well as proinsulin:insulin ratio decreased significantly between T1 and T2, while leptin concentrations and QUICKI value increased significantly.

Gestational age at birth and glucose-related hormone concentrations

Associations between gestational age at birth and glucose-related hormone concentrations at T1 and T2 are presented in online supplementary table 1. At T1, higher gestational age at birth was significantly associated with lower concentrations of glucose, ghrelin, and resistin, and higher concentrations of GLP-1. At T2, higher gestational age at birth was significantly

Confidential: For Review Only

associated with decreased concentrations of insulin and leptin, and increased proinsulin:insulin ratio. At both timepoints, higher gestational age at birth was significantly associated with lower concentrations of C-peptide and proinsulin, decreased HOMA2 index, and increased QUICKI value.

Glucose-related hormone concentrations and hyperglycemia

At T1, hyperglycemic infants (N=30) had significantly higher glucose and leptin concentrations than normoglycemic infants (N=18), as well as significantly lower QUICKI value (Figure 3). At T2, hyperglycemic infants (N=22) had significantly higher C-peptide and insulin concentrations than normoglycemic infants (N=14). At both timepoints, hyperglycemic infants had significantly higher proinsulin concentrations than normoglycemic infants.

Associations between glucose-related hormone concentrations at T1 and duration of subsequent hyperglycemia

Higher glucose and proinsulin concentrations and insulin:C-peptide ratio at T1, as well as lower QUICKI value, were significantly associated with more days with hyperglycemia > 10 mmol/L during the rest of the admission period (after T1; online supplementary table 2).

https://mc.manuscriptcentral.com/bmjpo

Confidential: For Review Only

Discussion

This prospective study reports concentrations of a broad range of glucose-related hormones in VLBW infants at two timepoints: DOL 7±3 days and PMA 36±1 weeks. Insulin resistance markers decreased while insulin sensitivity markers increased between the two timepoints. Prematurity was associated with higher concentrations of insulin resistance markers. Compared with normoglycemic infants, hyperglycemic infants had higher concentrations of a number of insulin resistance markers. Higher glucose and proinsulin concentrations and insulin:C-peptide ratio and lower QUICKI value at DOL 7 were associated with longer periods of hyperglycemia during the remaining admission period.

Glucose-related hormone concentrations reported in this study were higher than those reported in previous literature. For example, Salis et al. reported lower insulin, glucose, C-peptide and glucose concentrations, and higher GLP-1 concentrations and insulin:C-peptide ratio (28). However, substantial methodical differences (method of laboratory test, timing of blood sampling, population selection) render a comparison of such results difficult. No previous study has reported glucose-related hormone concentrations at precisely the same timepoints as the current study. Furthermore, the LIGHT-study included the largest proportion of extremely preterm infants compared with other published literature.

Concentrations of C-peptide, insulin, glucose, proinsulin, ghrelin, resistin and glucagon, as well as proinsulin:insulin ratio, have significantly decreased from DOL 7 to PMA 36 weeks, while leptin concentrations and QUICKI value significantly increased. This can be interpreted as an improvement in insulin resistance status, with a decrease in insulin resistance-promoting hormones and an increase in insulin sensitivity markers as the infant matures and the nutritional status improves. Unexpectedly, concentrations of GLP-1, an incretin hormone which is secreted in response to increased enteral nutrition and decreases insulin resistance,

Confidential: For Review Only

decreased with time. This might be due to the overall trend of decrease in insulin resistance with time which in turn would decrease the need in a GLP-1 response. These findings should be confirmed in future larger studies.

In our study, lower gestational age at birth was significantly associated with higher concentrations of glucose, ghrelin and resistin and lower concentrations of GLP-1 on DOL 7; with higher concentrations of insulin and leptin and decreased proinsulin:insulin ratio at PMA 36 weeks; and with higher concentrations of C-peptide and proinsulin, increased HOMA2 index and decreased QUICKI index at both timepoints. Overall, these results suggest an association between lower gestational age at birth and increased insulin resistance, both early in life and when nearing term age. Salis et al. have previously shown in a smaller study population that lower gestational age at birth was associated with higher insulin and glucose concentrations (25, 28).

Compared to normoglycemic infants, hyperglycemic infants had significantly higher concentrations of glucose and leptin and lower QUICKI value on DOL 7; higher concentrations of C-peptide and insulin at PMA 36 weeks; and higher concentrations of proinsulin at both timepoints. These results are supported by findings from two smaller previous studies (25, 27). This suggests that insulin resistance might be the mechanism responsible for hyperglycemia. Of note, there was no significant difference in proinsulin:insulin ratio between the groups, which does not support the theory of aberrant proinsulin processing as a cause of hyperglycemia (27). Another important finding is the relatively high HOMA2 scores noted in the different infant groups while mean glucose concentrations were not in the hyperglycemic range. This might suggest that VLBW infants have a subclinical insulin resistance even in the absence of overt hyperglycemia.

https://mc.manuscriptcentral.com/bmjpo

Confidential: For Review Only

The LIGHT-study also presents evidence for an association between glucose-related hormone concentrations and later hyperglycemia. Higher concentrations of glucose and proinsulin measured on DOL 7, as well as higher insulin:C-peptide ratio and lower QUICKI value (based on glucose and insulin concentrations) at the same timepoint, were significantly associated with higher number of days with hyperglycemia after this timepoint. This suggests that measuring these parameters at DOL 7 might help identify those infants that are most in need of close monitoring of glucose concentrations. Further studies are needed to explore the feasibility of using these markers to identify infants who have higher risk for prolonged hyperglycemia.

Only 25% of infants had quantifiable leptin concentrations on DOL 7 which increased to 92% of infants at PMA 36 weeks. Two previous studies in preterm infants have reported similar leptin concentrations in the first days of life (34, 35). Siahanido et al. on the other hand reported somewhat higher leptin concentrations at discharge (mean DOL 41) in preterm infants (36). The LIGHT-study included a higher proportion of extremely preterm infants which could explain the difference in the results, supporting in part the finding that more premature infants have increased insulin resistance and reflecting the poor nutritional status of these infants shortly after birth and its improvement during the admission period.

The LIGHT-study was a prospective study focused on glucose metabolism in VLBW infants. The many different hormonal pathways that affect glucose metabolism are intricately intertwined and this study is the first to report the results of a broad glucose-related hormone panel in this patient population. Furthermore, results are reported at two different timepoints, providing unique information regarding trends over time. To the best of our knowledge, it is the largest study to date to compare glucose-related hormone concentrations between hyperglycemic and normoglycemic infants. It is also the first study to report glucose-related

Confidential: For Review Only

hormone concentrations in preterm infants measured at the same predefined PMA nearing term age.

Although the largest to-date of its kind, the number of infants included in this study was relatively small, and larger studies are needed to confirm the findings. As the NICU at Umeå university hospital is one of six designated NICUs in Sweden providing care for extremely preterm infants, the LIGHT-study included a majority of extremely preterm infants (as opposed to VLBW). It should be noted that HOMA2 and QUICKI indeces, as well as the interpretation of glucose-related hormone concentrations in relation to one another in general, should usually be done using blood samples obtained in a fasting state. It would be unethical to implement this method in preterm infants and we have therefore used blood samples obtained just before enteral feedings which reflects the actual clinical situation in the NICU. Most of the infants received parenteral nutrition to some degree at T1, and two infants received parenteral nutrition at T2. This might have affected the concentrations measured to some extent. As mentioned earlier, the different hormonal pathways affecting glucose metabolism are complicated and difficult to isolate from one another. The results should therefore be interpreted with caution since it is debated whether insulin resistance markers (even in the fasting state) fully reflect the insulin resistance status in lean children.

Conclusion

VLBW infants seem to have a glucose-related hormone profile consistent with insulin resistance, both early in life and when nearing term age. Hyperglycemic preterm infants have higher concentrations of insulin resistance markers than normoglycemic infants. Lower gestational age is associated with higher concentrations of insulin resistance markers and insulin resistance decreases when nearing term age. Glucose and proinsulin concentrations,

Confidential: For Review Only

insulin:C-peptide ratio and QUICKI value might be useful markers to identify infants at risk for recurring hyperglycemia.

Acknowledgments

The authors would like to thank the participating parents and children, the medical staff in the neonatal units, and Lilla Barnets Fond for supporting this study.

Funding: The study was supported by research grants from Umeå University, Region Västerbotten, Lilla Barnets Fond, Samariten Foundation, Oskar Foundation and Arnerska Research Foundation. No involvement was made by these organizations in the study design; the collection, analysis, and interpretation of data; the writing of the report; and the decision to submit the paper for publication.

References

1. Pertierra-Cortada A, Ramon-Krauel M, Iriondo-Sanz M, Iglesias-Platas I. Instability of glucose values in very preterm babies at term postmenstrual age. J Pediatr. 2014;165(6):1146-53 e2.
2. Mola-Schenzle E, Staffler A, Klemme M, Pellegrini F, Molinaro G, Parhofer KG, et al. Clinically stable very low birthweight infants are at risk for recurrent tissue glucose fluctuations even after fully established enteral nutrition. Arch Dis Child Fetal Neonatal Ed. 2015;100(2):F126-31.
3. Zamir I, Tornevi A, Abrahamsson T, Ahlsson F, Engstrom E, Hallberg B, et al. Hyperglycemia in Extremely Preterm Infants-Insulin Treatment, Mortality and Nutrient Intakes. J Pediatr. 2018;200:104-10 e1.
4. Hays SP, Smith EO, Sunehag AL. Hyperglycemia is a risk factor for early death and morbidity in extremely low birth-weight infants. Pediatrics. 2006;118(5):1811-8.
5. Zamir I, Stoltz Sjostrom E, Ahlsson F, Hansen-Pupp I, Serenius F, Domellof M. Neonatal hyperglycaemia is associated with worse neurodevelopmental outcomes in extremely preterm infants. Arch Dis Child Fetal Neonatal Ed. 2021;106(5):460-6.
6. Zamir I, Stoltz Sjostrom E, Edstedt Bonamy AK, Mohlkert LA, Norman M, Domellof M. Postnatal nutritional intakes and hyperglycemia as determinants of blood pressure at 6.5 years of age in children born extremely preterm. Pediatr Res. 2019;86(1):115-21.
7. Auerbach A, Eventov-Friedman S, Arad I, Peleg O, Bdolah-Abram T, Bar-Oz B, et al. Long duration of hyperglycemia in the first 96 hours of life is associated with severe intraventricular hemorrhage in preterm infants. J Pediatr. 2013;163(2):388-93.
8. Alexandrou G, Skiold B, Karlen J, Tessma MK, Norman M, Aden U, et al. Early hyperglycemia is a risk factor for death and white matter reduction in preterm infants. Pediatrics. 2010;125(3):e584-91.
9. Mohsen L, Abou-Alam M, El-Dib M, Labib M, Elsada M, Aly H. A prospective study on hyperglycemia and retinopathy of prematurity. J Perinatol. 2014;34(6):453-7.
10. Ramel SE, Long JD, Gray H, Durrwachter-Erno K, Demerath EW, Rao R. Neonatal hyperglycemia and diminished long-term growth in very low birth weight preterm infants. J Perinatol. 2013;33(11):882-6.
11. Hamayun J, Mohlkert LA, Stoltz Sjostrom E, Domellof M, Norman M, Zamir I. Association between Neonatal Intakes and Hyperglycemia, and Left Heart and Aortic Dimensions at 6.5 Years of Age in Children Born Extremely Preterm. J Clin Med. 2021;10(12).
12. Hofman PL, Regan F, Jackson WE, Jefferies C, Knight DB, Robinson EM, et al. Premature birth and later insulin resistance. N Engl J Med. 2004;351(21):2179-86.

Confidential: For Review Only

13. Balasuriya CND, Stunes AK, Mosti MP, Schei B, Indredavik MS, Hals IK, et al. Metabolic Outcomes in Adults Born Preterm With Very Low Birthweight or Small for Gestational Age at Term: A Cohort Study. J Clin Endocrinol Metab. 2018;103(12):4437-46.
14. Bo S, Bertino E, Bagna R, Trapani A, Gambino R, Martano C, et al. Insulin resistance in pre-school very-low-birth weight pre-term children. Diabetes Metab. 2006;32(2):151-8.
15. de Jong M, Cranendonk A, van Weissenbruch MM. Components of the metabolic syndrome in early childhood in very-low-birth-weight infants and term small and appropriate for gestational age infants. Pediatr Res. 2015;78(4):457-61.
16. de Mendonca E, de Lima Macena M, Bueno NB, de Oliveira ACM, Mello CS. Premature birth, low birth weight, small for gestational age and chronic non-communicable diseases in adult life: A systematic review with meta-analysis. Early Hum Dev. 2020;149:105154.
17. Markopoulou P, Papanikolaou E, Analytis A, Zoumakis E, Siahanidou T. Preterm Birth as a Risk Factor for Metabolic Syndrome and Cardiovascular Disease in Adult Life: A Systematic Review and Meta-Analysis. J Pediatr. 2019;210:69-80 e5.
18. Mathai S, Cutfield WS, Derraik JG, Dalziel SR, Harding JE, Robinson E, et al. Insulin sensitivity and beta-cell function in adults born preterm and their children. Diabetes. 2012;61(10):2479-83.
19. Morrison KM, Ramsingh L, Gunn E, Streiner D, Van Lieshout R, Boyle M, et al. Cardiometabolic Health in Adults Born Premature With Extremely Low Birth Weight. Pediatrics. 2016;138(4).
20. Regan FM, Cutfield WS, Jefferies C, Robinson E, Hofman PL. The impact of early nutrition in premature infants on later childhood insulin sensitivity and growth. Pediatrics. 2006;118(5):1943-9.
21. Rotteveel J, van Weissenbruch MM, Twisk JW, Delemarre-Van de Waal HA. Infant and childhood growth patterns, insulin sensitivity, and blood pressure in prematurely born young adults. Pediatrics. 2008;122(2):313-21.
22. Pollak A, Cowett RM, Schwartz R, Oh W. Glucose disposal in low-birth-weight infants during steady-state hyperglycemia: effects of exogenous insulin administration. Pediatrics. 1978;61(4):546-9.
23. Blanco CL, McGill-Vargas LL, Gastaldelli A, Seidner SR, McCurnin DC, Leland MM, et al. Peripheral insulin resistance and impaired insulin signaling contribute to abnormal glucose metabolism in preterm baboons. Endocrinology. 2015;156(3):813-23.
24. McGill-Vargas L, Gastaldelli A, Liang H, Anzueto Guerra D, Johnson-Pais T, Seidner S, et al. Hepatic Insulin Resistance and Altered Gluconeogenic Pathway in Premature Baboons. Endocrinology. 2017;158(5):1140-51.
25. Salis ER, Reith DM, Wheeler BJ, Broadbent RS, Medlicott NJ. Hyperglycaemic preterm neonates exhibit insulin resistance and low insulin production. BMJ Paediatr Open. 2017;1(1):e000160.
26. Bansal A, Bloomfield FH, Connor KL, Dragunow M, Thorstensen EB, Oliver MH, et al. Glucocorticoid-Induced Preterm Birth and Neonatal Hyperglycemia Alter Ovine beta-Cell Development. Endocrinology. 2015;156(10):3763-76.
27. Mitanchez-Mokhtari D, Lahlou N, Kieffer F, Magny JF, Roger M, Voyer M. Both relative insulin resistance and defective islet beta-cell processing of proinsulin are responsible for transient hyperglycemia in extremely preterm infants. Pediatrics. 2004;113(3 Pt 1):537-41.
28. Salis ER, Reith DM, Wheeler BJ, Broadbent RS, Medlicott NJ. Insulin resistance, glucagon-like peptide-1 and factors influencing glucose homeostasis in neonates. Arch Dis Child Fetal Neonatal Ed. 2017;102(2):F162-F6.
29. Lucas A, Bloom SR, Aynsley-Green A. Metabolic and endocrine events at the time of the first feed of human milk in preterm and term infants. Arch Dis Child. 1978;53(9):731-6.
30. Asplund K. Effects of postnatal feeding on the functional maturation of pancreatic islet B-cells of neonatal rats. Diabetologia. 1972;8(3):153-9.
31. Cowett RM, Oh W, Schwartz R. Persistent glucose production during glucose infusion in the neonate. J Clin Invest. 1983;71(3):467-75.
32. Sunehag A, Gustafsson J, Ewald U. Very immature infants (< or = 30 Wk) respond to glucose infusion with incomplete suppression of glucose production. Pediatr Res. 1994;36(4):550-5.

Confidential: For Review Only

33. Ogilvy-Stuart AL, Beardsall K. Management of hyperglycaemia in the preterm infant. Arch Dis Child Fetal Neonatal Ed. 2010;95(2):F126-31.
34. Park MJ, Namgung R, Kim JN, Kim DH. Serum leptin, IGF-I and insulin levels in preterm infants receiving parenteral nutrition during the first week of life. J Pediatr Endocrinol Metab. 2001;14(4):429-33.
35. Ng PC, Lam CW, Lee CH, Wong GW, Fok TF, Wong E, et al. Changes of leptin and metabolic hormones in preterm infants: a longitudinal study in early postnatal life. Clin Endocrinol (Oxf). 2001;54(5):673-80.
36. Siahanidou T, Mandyla H, Papassotiriou GP, Papassotiriou I, Chrousos G. Circulating levels of adiponectin in preterm infants. Arch Dis Child Fetal Neonatal Ed. 2007;92(4):F286-90.

Confidential: For Review Only

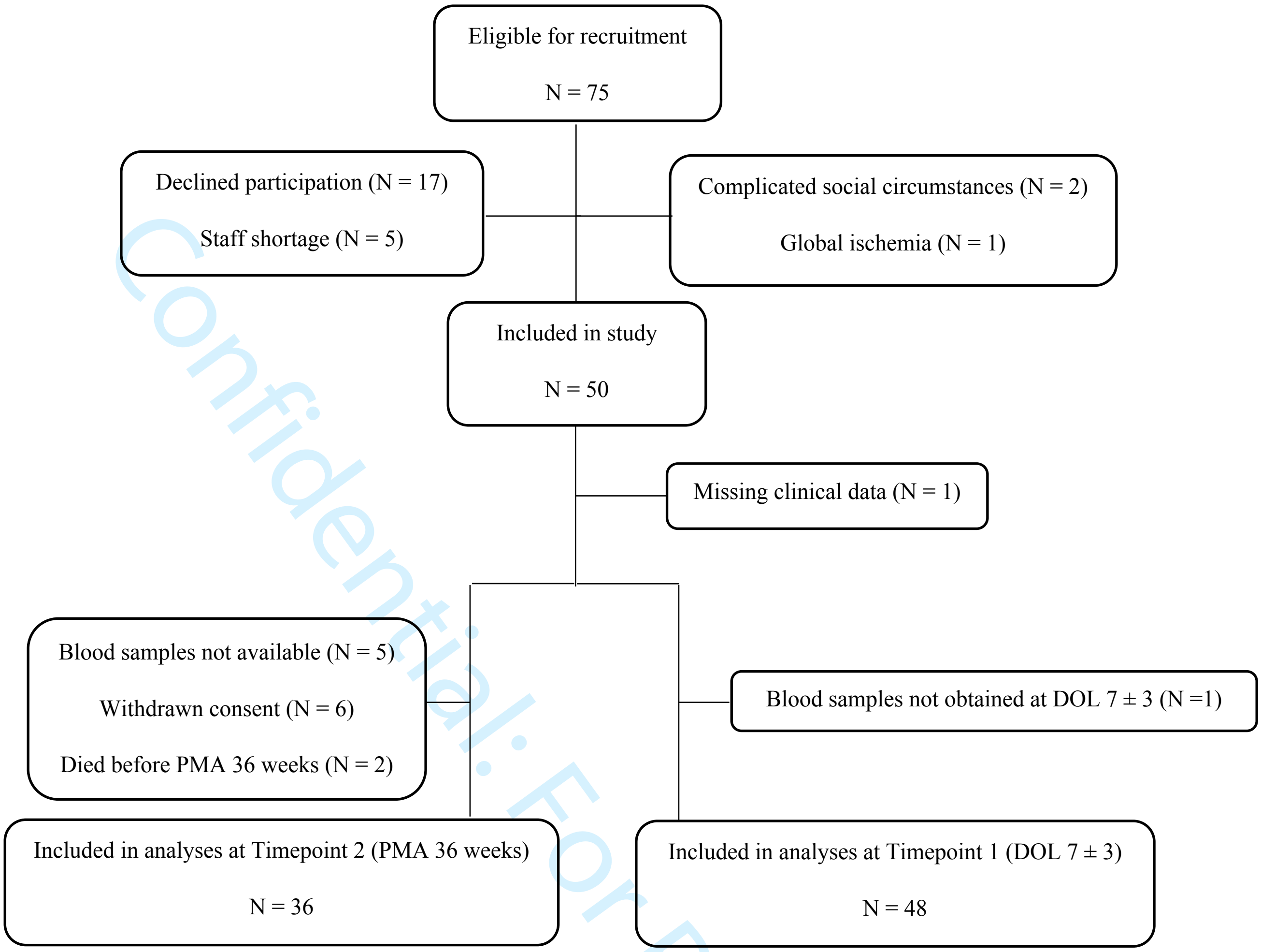

Figure 1. Included and excluded very low birth weight infants. PMA – postmenstrual age; DOL – day of life.

Confidential: For Review Only

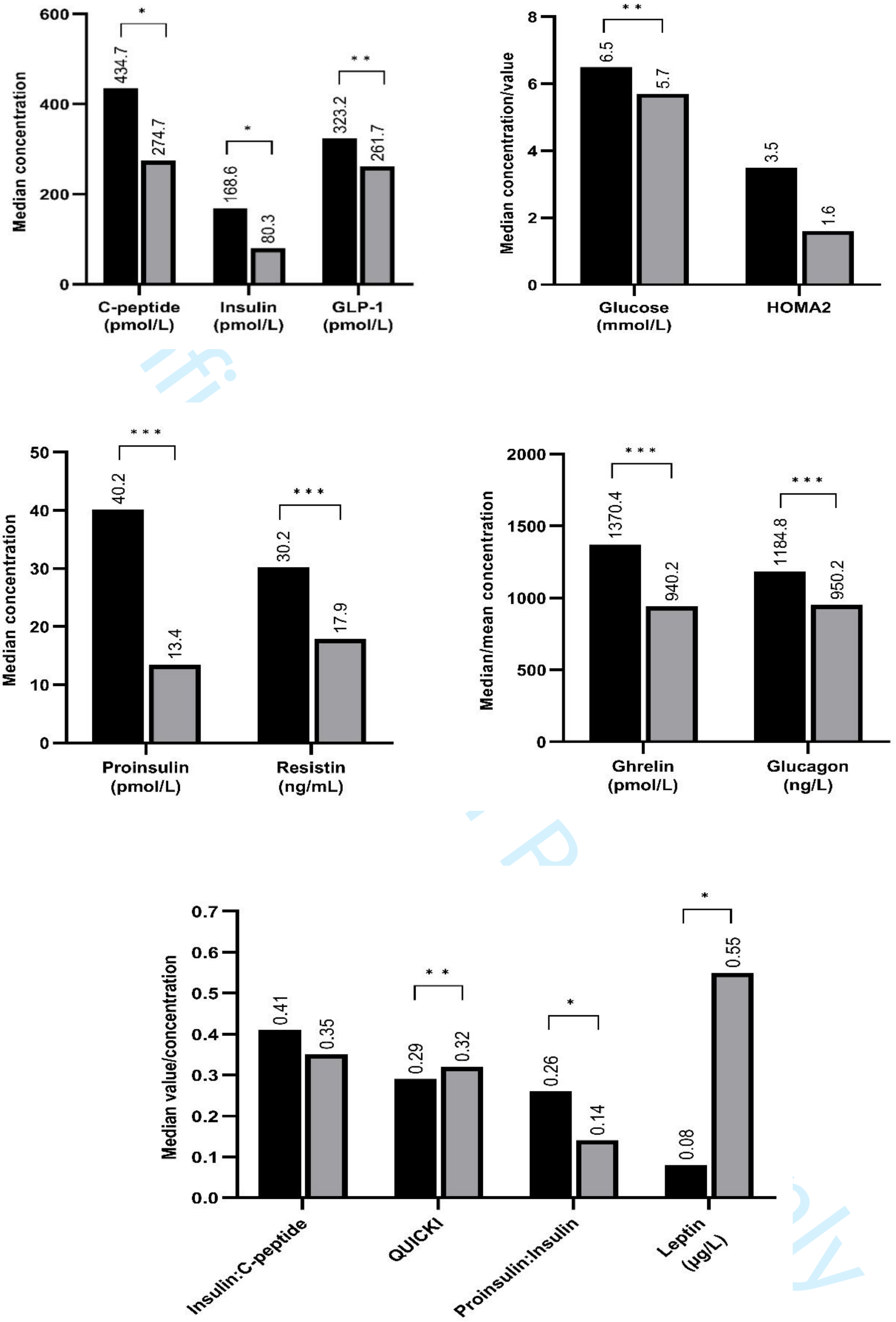

Figure 2. Concentrations of glucose-related hormones in very low birth weight infants on day of life 7±3 (Timepoint 1; black) compared to concentrations at postmenstrual age 36±1 weeks (Timepoint 2; grey). * P<0.05; ** P<0.01; *** P<0.001. GLP-1 – glucagon-like peptide 1; HOMA2 – homeostatic model assessment 2; QUICKI – quantitative insulin sensitivity check index.

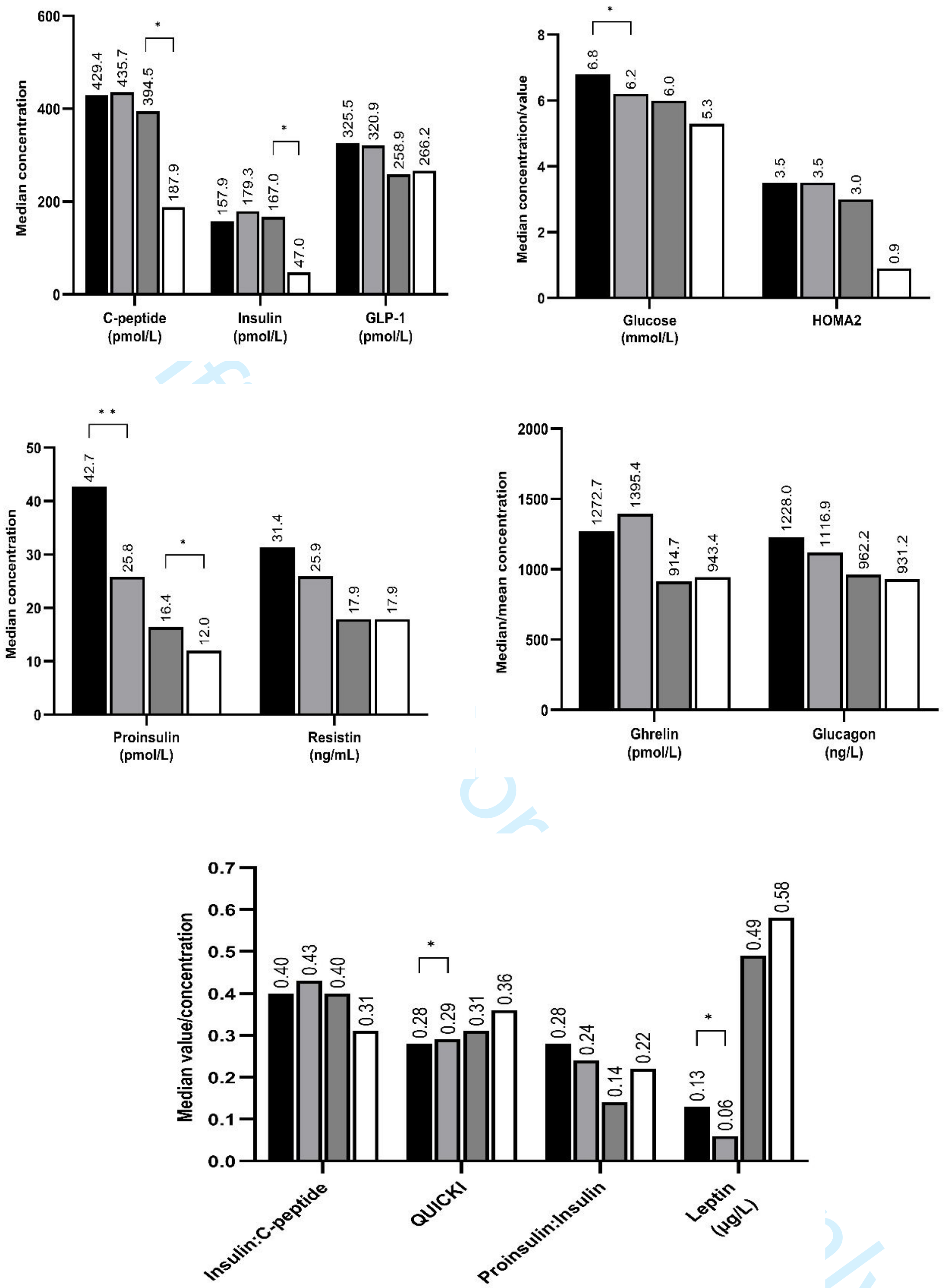

Figure 3. Concentrations of glucose-related hormones in hyperglycemic and normoglycemic very low birth weight infants sampled on day of life 7±3 (Timepoint 1; black and light grey, respectively) and at postmenstrual age 36±1 weeks (Timepoint 2; dark grey and white, respectively). * P<0.05; ** P<0.01; *** P<0.001. GLP-1 – glucagon-like peptide 1; HOMA2 – homeostatic model assessment 2; QUICKI – quantitative insulin sensitivity check index.

505 **Table 1. Cohort characteristics.**

Characteristic	DOL 7 (Timepoint 1) N=48	PMA 36 weeks (Timepoint 2) N=36
**Perinatal variables**		
Gestational age, weeks, mean (SD)	27.2 (2.5)	27.3 (2.7)
Extremely preterm (< 28 weeks), N (%)	31 (65)	23 (64)
Male sex, N (%)	14 (29)	11 (31)
Multiple gestation, N (%)	6 (13)	3 (8)
Small for gestational age*, N (%)	20 (42)	16 (44)
Antenatal steroid treatment, N (%)	44 (92)	34 (94)
Amnionitis, N (%)	4 (8)	4 (11)
Preeclampsia, N (%)	11 (23)	8 (22)
Cesarean section, N (%)	36 (75)	27 (75)
Apgar 1 min, mean (SD)	5 (3)	5 (2)
Apgar 5 min, mean (SD)	7 (2)	8 (2)**
Apgar 10 min, mean (SD)	8 (2)	8 (1)
**Anthropometric variables at birth**		
Weight, grams, mean (SD)	936 (280)	925 (296)
Weight z-score, mean (SD)	-1.27 (1.52)	-1.51 (1.61)
Length, cm, mean (SD)	34.7 (3.4)	34.6 (3.7)
Length z-score, mean (SD)	-1.69 (1.91)	-1.88 (2.03)
Head circumference, cm, mean (SD)	24.4 (2.4)	24.4 (2.5)
Head circumference z-score, mean (SD)	-0.95 (0.98)	-1.05 (1.05)

Confidential: For Review Only

**Neonatal variables**		
Duration (in days) of mechanical ventilation treatment during admission period, mean (SD)	8.9 (13.6)	8.7 (13.3)
Inotrope treatment, N (%)	7 (15)	4 (11)
Treatment with systemic steroids, N (%)	11 (23)	8 (22)
Insulin treatment, N (%)	9 (19)	7 (19)
Culture-verified sepsis during admission period, N (%)	5 (10)	4 (11)
Intraventricular hemorrhage		
None, N (%)	35 (73)	26 (72)
Grade 1-2, N (%)	7 (15)	6 (17)
Grade 3-4, N (%)	6 (13)	4 (11)
Necrotizing enterocolitis, N (%)	1 (2)	0 (0)
Patent ductus arteriosus, N (%)	29 (60)	18 (50)**
Periventricular leukomalacia, N (%)	1 (2)	1 (3)
Retinopathy of prematurity		
None, N (%)	30 (63)	22 (61)
Stage 1-2, N (%)	9 (19)	6 (17)
Stage 3-5, N (%)	9 (19)	8 (22)
Bronchopulmonary dysplasia, N (%)	19 (40) (N = 47)	16 (44)

* defined as birth weight < 10th %-ile; ** P < 0.05 when comparing infants remaining in the study and excluded infants at Timepoint 2. DOL – day of life; PMA – postmenstrual age.

Confidential: For Review Only

Table 2. Concentrations of glucose-related hormones in very low birth weight infants at day of life 7±3 (Timepoint 1) and at postmenstrual age 36±1 weeks (Timepoint 2).

	**Timepoint 1**<br><br>**N=48**	**Timepoint 2**<br><br>**N=36**
C-peptide, pmol/L, median (IQR)	440.5 (253.6-618.8)	274.7 (156.9-537.6)
Insulin, pmol/L, median (IQR)	183.8 (108.7-288.4)	80.3 (39.6-202.3)
Insulin:C-peptide, median (IQR)	0.42 (0.34-0.55)	0.35 (0.25-0.47)
Glucose, mmol/L, median (IQR)	6.5 (5.7-8.5)	5.7 (4.9-6.7)
HOMA2, median (IQR)	3.6 (2.1-5.8)	1.6 (0.8-3.8)
QUICKI, median (IQR)	0.28 (0.27-0.30)	0.32 (0.29-0.36)
Proinsulin, pmol/L, median (IQR)	42.6 (31.5-61.8)	13.4 (10.0-21.0)
Proinsulin:insulin, median (IQR)	0.27 (0.19-0.34)	0.14 (0.09-0.30)
Leptin, µg/L, median (IQR)*	0.11 (0.05-0.25)	0.55 (0.33-1.18)
Ghrelin, pmol/L, median (IQR)	1326.3 (1029.0-1694.8)	940.2 (826.9-1110.2)
GLP1, pmol/L, median (IQR)	318.3 (230.7-428.9)	261.7 (194.6-366.1)
Resistin, ng/mL, median (IQR)	29.9 (24.8-36.2)	17.9 (15.1-25.3)
Glucagon, ng/L, mean (SD)	1175.1 (264.6)	950.2 (254.0)

* Quantifiable concentrations of leptin were found in 12 infants (25%) at Timepoint 1 and in 33 infants (92%) at Timepoint 2 (Lower limit of quantification 0.01 mcg/L). IQR – interquartile range; HOMA2 – homeostatic model assessment 2; QUICKI – quantitative insulin sensitivity check index; GLP-1 – glucagon-like peptide.

Confidential: For Review Only

Online supplementary table 1. Associations between gestational age at birth and glucose-related hormone concentrations on day of life 7±3 (Timepoint 1) and at postmenstrual age 36±1 weeks (Timepoint 2) in very low birth weight infants.

	**Timepoint 1**			**Timepoint 2**		
	Coefficient	95% CI	P value	Coefficient	95% CI	P value
C-peptide	-39.7	-72.3 to -7.2	**0.02**	-51.8	-81.8 to -21.9	**0.001**
Insulin	-16.6	-34.3 to 1.2	0.07	-29.9	-49.0 to -10.7	**0.003**
Insulin: C-peptide	-0.01	-0.03 to 0.01	0.43	-0.02	-0.04 to 0.01	0.13
Glucose	-0.4	-0.7 to -0.2	**0.003**	-0.2	-0.4 to 0.01	0.07
HOMA2	-0.4	-0.7 to -0.03	**0.03**	-0.7	-1.1 to -0.3	**0.001**
QUICKI	0.006	0.002 to 0.01	**0.002**	0.012	0.005 to 0.018	**0.001**
Proinsulin	-6.1	-8.4 to -3.8	**< 0.001**	-1.4	-2.5 to -0.3	**0.01**
Proinsulin: Insulin	-0.01	-0.05 to 0.02	0.44	0.02	0.00 to 0.04	**0.048**
Leptin*	-0.06	-0.29 to 0.16	0.55	-0.15	-0.2 to -0.07	**0.001**
Ghrelin	-79.0	-141.2 to -16.8	**0.01**	-1.8	-62.1 to 58.4	0.95
GLP-1	20.1	0.1 to 40.1	**0.049**	1.8	-14.5 to 18.2	0.82
Resistin	-1.6	-2.6 to -0.6	**0.002**	-0.1	-1.0 to 0.9	0.88

Confidential: For Review Only

Glucagon	-4.4	-37.7 to 29.0	0.79	12.3	-20.8 to 45.4	0.45

* Quantifiable concentrations of leptin were found in 12 infants (25%) at Timepoint 1 and in 33 infants (92%) at Timepoint 2 (LLOQ 0.01 mcg/L). HOMA2 – homeostatic model assessment 2; QUICKI – quantitative insulin sensitivity check index; GLP-1 – glucagon-like peptide.

Confidential: For Review Only

540 **Online supplementary table 2. Associations between glucose-related hormone**
541 **concentrations on day of life 7±3 (Timepoint 1) and duration of subsequent**
542 **hyperglycemia in very low birth weight infants.**

**Glucose-related parameter (increase in value measured at Timepoint 1)**	**N days with hyperglycemia after Timepoint 1**			
	B	95% CI	P value	R^2
C-peptide (+100 pmol/L)	0.1	-0.5 to 0.7	0.68	0.004
Insulin (+100 pmol/L)	0.7	-0.3 to 1.8	0.18	0.04
Insulin:C-peptide (+0.1)	1.48	0.69 to 2.28	**< 0.001**	0.24
Glucose (+1 mmol/L)	1.26	0.71 to 1.81	**< 0.001**	0.32
HOMA (+1)	0.48	-0.06 to 1.02	0.08	0.07
QUICKI (+0.01)	-0.48	-0.91 to -0.05	**0.03**	0.08
Proinsulin (+10 pmol/L)	0.7	0.03 to 1.4	**0.04**	0.09
Proinsulin:insulin (+0.1)	0.03	-0.56 to 0.62	0.92	0.0
Leptin (+0.1 mcg/L)	-0.06	-0.64 to 0.51	0.82	0.001
Ghrelin (+100 pmol/L)	0.2	-0.04 to 0.5	0.09	0.06
GLP-1 (+1 pmol/L)	0.0	-0.01 to 0.01	0.95	0.0
Resistin (+1 ng/mL)	0.11	-0.08 to 0.29	0.25	0.03
Glucagon (+100 ng/L)	0.1	-0.5 to 0.7	0.84	0.001

543 HOMA2 – homeostatic model assessment 2; QUICKI – quantitative insulin sensitivity check
544 index; GLP-1 – glucagon-like peptide.

Confidential: For Review Only

BMJ Paediatrics Open

Insulin resistance prior to term age in very low birth infants: a prospective study

Journal:	*BMJ Paediatrics Open*
Manuscript ID	bmjpo-2023-002470.R1
Article Type:	Original research
Date Submitted by the Author:	23-Jan-2024
Complete List of Authors:	Zamir, Itay; Umeå University, Department of Clinical Sciences, Pediatrics<br>Stoltz Sjöström, Elisabeth; Umeå University, Department of Food, Nutrition and Culinary Science<br>van Den Berg, Johannes; Umeå University, Department of Clinical Sciences, Pediatrics<br>Naumburg, Estelle; Umeå University, Department of Clinical Sciences, Pediatrics<br>Domellof, Magnus; Umeå University, Department of Clinical Sciences, Pediatrics
Keywords:	Endocrinology, Neonatology

SCHOLARONE™
Manuscripts

I, the Submitting Author has the right to grant and does grant on behalf of all authors of the Work (as defined in the below author licence), an exclusive licence and/or a non-exclusive licence for contributions from authors who are: i) UK Crown employees; ii) where BMJ has agreed a CC-BY licence shall apply, and/or iii) in accordance with the terms applicable for US Federal Government officers or employees acting as part of their official duties; on a worldwide, perpetual, irrevocable, royalty-free basis to BMJ Publishing Group Ltd ("BMJ") its licensees and where the relevant Journal is co-owned by BMJ to the co-owners of the Journal, to publish the Work in this journal and any other BMJ products and to exploit all rights, as set out in our licence.

The Submitting Author accepts and understands that any supply made under these terms is made by BMJ to the Submitting Author unless you are acting as an employee on behalf of your employer or a postgraduate student of an affiliated institution which is paying any applicable article publishing charge ("APC") for Open Access articles. Where the Submitting Author wishes to make the Work available on an Open Access basis (and intends to pay the relevant APC), the terms of reuse of such Open Access shall be governed by a Creative Commons licence – details of these licences and which Creative Commons licence will apply to this Work are set out in our licence referred to above.

Other than as permitted in any relevant BMJ Author's Self Archiving Policies, I confirm this Work has not been accepted for publication elsewhere, is not being considered for publication elsewhere and does not duplicate material already published. I confirm all authors consent to publication of this Work and authorise the granting of this licence.

Confidential: For Review Only

Insulin resistance prior to term age in very low birth infants: a prospective study

Itay Zamir, MD, PhD1, Elisabeth Stoltz Sjöström, RD, PhD2, Johannes van Den Berg, RN, PhD1, Estelle Naumburg, MD, PhD1, and Magnus Domellöf, MD, PhD1.

Affiliations:
1 Department of Clinical Sciences, Pediatrics, Umeå University, Umeå, Sweden.
2 Department of Food, Nutrition and Culinary Science, Umeå University, Umeå, Sweden.

Address correspondence to: Itay Zamir, Department of Clinical Sciences, Pediatrics, Umeå University, SE-90187 Umeå, Sweden, itay.zamir@umu.se, Orcid-id: 0000-0001-9086-7991

Abbreviations:
DOL – day of life
GIP – gastric inhibitory polypeptide
GLP-1 – glucagon-like peptide 1
HOMA2 – homeostatic model assessment 2
LIGHT – very low birth weight infants, glucose and hormonal profiles over time
LLOQ – lower limit of quantification
NICU – neonatal intensive care unit
PMA – postmenstrual age
QUICKI – Quantitative insulin sensitivity check index
VLBW – very low birth weight

Keywords: very low birth weight; hyperglycemia; insulin resistance; insulin

Confidential: For Review Only

Abstract

Objective: To explore the glucose-related hormone profile of very low birth weight (VLBW) infants and assess the association between neonatal hyperglycemia and insulin resistance during the admission period.

Design: A prospective observational study – the very low birth weight infants, glucose and hormonal profiles over time (LIGHT) study.

Setting: A tertiary neonatal intensive care unit (NICU) and four neonatal units in county hospitals in Sweden.

Patients: 48 infants born <1500 grams (VLBW) during 2016-2019.

Outcome measures: Plasma concentrations of glucose-related hormones and proteins (C-peptide, insulin, ghrelin, glucagon-like peptide 1 (GLP-1), glucagon, leptin, resistin, and proinsulin), insulin:C-peptide and proinsulin:insulin ratios, Homeostatic Model Assessment (HOMA2) and Quantitative Insulin Sensitivity Check (QUICKI) indeces, measured on day of life (DOL) 7 and at postmenstrual age 36 weeks.

Results: Lower gestational age was significantly associated with higher glucose, C-peptide, insulin, proinsulin, leptin, ghrelin, resistin and GLP-1 concentrations, increased HOMA2 index, and decreased QUICKI index and proinsulin:insulin ratio. Hyperglycemic infants had significantly higher glucose, C-peptide, insulin, leptin and proinsulin concentrations, and lower QUICKI index, than normoglycemic infants. Higher glucose and proinsulin concentrations and insulin:C-peptide ratio, and lower QUICKI index on DOL 7 were significantly associated with longer duration of hyperglycemia during the admission period.

Confidential: For Review Only

Conclusions: VLBW infants seem to have a hormone profile consistent with insulin resistance. Lower gestational age and hyperglycemia are associated with higher concentrations of insulin resistance markers.

What is already known on this topic:

- Hyperglycemia, a condition associated with many detrimental complications, is common in preterm infants during the postnatal period but its cause is debated.
- Children born preterm are more prone to develop insulin resistance during their lifetime.

What this study adds:

- VLBW infants seem to have laboratory signs of insulin resistance early in life, already during the admission period at the NICU, with hyperglycemic infants having higher levels of insulin resistance markers than normoglycemic infants.

How this study might affect research, practice or policy:

- The study supplies data regarding hormone levels in preterm infants and markers that might be associated with hyperglycemia later during the admission period, which might be of use for clinicians.
- The study reports data supporting the theory that the mechanism of neonatal hyperglycemia is related to increased insulin resistance, which might be useful for future research of possible treatment methods.

Confidential: For Review Only

Introduction

Hyperglycemia is common in preterm born infants. Up to 80% of extremely preterm infants (< 28 gestational weeks) and very low birth weight infants (VLBW; < 1500 g) are exposed to hyperglycemia during the first week of life, and the condition is common later during the admission period as well(1-3). Hyperglycemia is also associated with a multitude of adverse outcomes, including increased mortality and impaired neurodevelopment(3-11). It is well known that children born preterm have increased risk of insulin resistance and metabolic syndrome later in life(12-21).

The mechanisms responsible for hyperglycemia in the preterm infant are not fully understood. Reduced peripheral insulin sensitivity has been suggested as a possible mechanism(22-25). Another possible explanation suggested is beta cell dysfunction, either due to reduced beta cell mass in the pancreas(26) or aberrant processing of proinsulin in these cells which leads to relative insulin deficiency(27). A deficient incretin response due to late onset of enteral feeds(28-30), and insufficient control of glucose production are postulated mechanisms as well(31, 32). Other perinatal clinical conditions, such as sepsis, might be involved in the pathophysiology of hyperglycemia(33).

Very few studies have reported hormone concentrations in preterm infants and more information is needed to clarify the mechanisms causing hyperglycemia in these infants.

This study aimed at investigating the glucose-related hormone profile of VLBW infants during the admission period at the neonatal intensive care unit (NICU), including possible associations with hyperglycemia.

Confidential: For Review Only

Methods

Study population

A prospective cohort study - The Very Low Birth Weight Infants, Glucose and Hormonal Profiles over Time (LIGHT) - included VLBW infants admitted to the NICU at the tertiary university hospital in Umeå, Sweden, between October 1, 2016, and November 30, 2019. Infants were included within the first week after birth if they were transported to the NICU within 24 hours after birth, remained at the NICU for the first week of life, did not have congenital malformations or chromosomal aberrations, and were registered at the four participating county hospitals.

Seventy-five infants were eligible for recruitment. Of these, 67 were approached (8 were not approached due to staff shortage, complicated social circumstances, and extensive brain injury in the infant) and 50 (75%) approved participation (Figure 1). Two infants (4%) died before reaching postmenstrual age (PMA) 36 weeks and six infants (12%) left the study due to withdrawal of consent before reaching this timepoint.

Study procedure

Blood samples (0.6 mL whole blood) were obtained from the infants on day of life (DOL) 7±3 days (Timepoint 1; T1) and at PMA 36±1 weeks (Timepoint 2; T2). The samples were obtained just before enteral feedings, together with clinically indicated blood samples. Parenteral nutrition was administered to some extent in 44 infants at T1 and in 2 infants at T2 at the time of sampling. No infant was receiving inotrope or corticosteroid treatment when the samples were obtained. All blood sampled at T1 were obtained at the NICU in Umeå while blood samples at T2 were obtained at all participating hospitals. When possible, topical anesthetics were used prior to venipuncture. After a coagulation time of approximately one

https://mc.manuscriptcentral.com/bmjpo
Confidential: For Review Only

hour, the samples were centrifuged, and serum was separated into two 150 µL tubes. The tubes were immediately stored in a freezer at -80° C. No protease inhibitor was added.

One infant (2%) was excluded from all analyses due to missing clinical data (Figure 1). One infant (2%) was excluded from analyses regarding T1 due to blood sample obtained out of given timeframe. Thus, blood samples at T1 from 48 infants (96%) were included in the analyses. Samples from 41 infants (82%) were obtained at T2, of which 5 samples were not available for analysis. Thus, blood samples at T2 from 36 infants (72%) were included in the analyses. In five of them, glucose concentrations were not obtained simultaneously as blood sampling and these infants were thus excluded from analyses involving glucose concentrations.

Glucose-related hormone profile

Serum samples were thawed and analyzed for concentrations of C-peptide, insulin, ghrelin, gastric inhibitory polypeptide (GIP), glucagon-like peptide 1 (GLP-1), glucagon, leptin, resistin and visfatin using Bio-Plex® Pro human diabetes multiplex immunoassay (Bio-Rad Laboratories, Inc. Hercules, California). Interassay variation was 1-6% for all analytes except ghrelin (15%), and intraassay variation was below 15%. Proinsulin concentrations were measured using an enzyme-linked immunosorbent assay (EMD Millipore Corp. St. Louis, Missouri). Interassay variation was 4% and intraassay variation was 1-9%.

Insulin:C-peptide and proinsulin:insulin ratios were calculated. Homeostatic Model assessment 2 (HOMA2) index was calculated using a calculator released by the Diabetes Trials Unit, University of Oxford: HOMA Calculator. The calculator is available at: http://www.dtu.ox.ac.uk/homacalculator/index.php (version 2.2.4, updated November 27, 2019). Quantitative insulin sensitivity check index (QUICKI) was calculated using the formula:

Confidential: For Review Only

$$1/(\log(insulin\ \mu U/mL) + \log(glucose\ mg/dL))$$

Perinatal data

Comprehensive perinatal data, including daily glucose values and neonatal morbidities, were collected prospectively from medical notes and are listed in Table 1. Hyperglycemia was defined as any single glucose concentration >10 mmol/L(5). Local guidelines at the NICU recommend starting insulin infusion if glucose concentration is above 13 mmol/L and decreasing glucose intake has not remedied the hyperglycemia. No sample analyzed for glucose-related hormone concentrations was taken while insulin treatment was ongoing.

Statistical analyses

The analyses were done using SPSS Statistical software (IBM corp. Released 2020. IBM SPSS Statistics for Windows, Version 27.0. Armonk, NY: IBM Corp.). Student t-test and Mann-Whitney U-test with calculations of standard deviations and interquartile ranges were used as appropriate. Generalized linear mixed models with a linear link function were used to assess associations between gestational age at birth and concentrations of glucose-related hormones at T1 and T2. Linear regression was used to evaluate the associations between concentrations of glucose-related hormones at T1 and number of days with hyperglycemia afterwards (rest of the admission period). The significance level was set to $P<0.05$.

Ethical approval

The study was approved by the regional ethical committee in Umeå, Sweden (Dnr 226-31M/2016), and written informed consent was obtained from all legal guardians.

Patient and public involvement

Confidential: For Review Only

Patients or the public were not involved in the design, or conduct, or reporting, or dissemination plans of our research.

Confidential: For Review Only

Results

Cohort characteristics and anthropometric data are presented in Table 1. Statistically significant differences between infants included at T1 (DOL 7±3 days) and T2 (PMA 36±1 weeks) included higher Apgar scores at 5 min and lower prevalence of patent ductus arteriosus in the infants analyzed at T2.

Concentrations of glucose-related hormones

Concentrations of glucose-related hormones at T1 and T2 in the entire cohort are presented in Table 2. Quantifiable concentrations of visfatin were found only in one infant (2%) at T1 and in none (0%) at T2 (Lower limit of quantification (LLOQ) 35.6 pg/mL) and are thereforbe not presented. Quantifiable concentrations of GIP were found only in one infant (2%) at T1 and in six infants (17%) at T2 (LLOQ 8.5 pmol/L), which was deemed to be too few samples to proceed with statical analyses.

To evaluate change in glucose-related hormone concentrations over time, we compared concentrations between infants for whom samples were available at both timepoints (N=36; N=7 for leptin). The results are presented in Figure 2. Concentrations of C-peptide, insulin, glucose, proinsulin, ghrelin, GLP-1, resistin and glucagon as well as proinsulin:insulin ratio decreased significantly between T1 and T2, while leptin concentrations and QUICKI value increased significantly.

Gestational age at birth and glucose-related hormone concentrations

Associations between gestational age at birth and glucose-related hormone concentrations at T1 and T2 are presented in supplementary file 1. At T1, higher gestational age at birth was significantly associated with lower concentrations of glucose, ghrelin, and resistin, and higher concentrations of GLP-1. At T2, higher gestational age at birth was significantly associated

Confidential: For Review Only

with decreased concentrations of insulin and leptin, and increased proinsulin:insulin ratio. At both timepoints, higher gestational age at birth was significantly associated with lower concentrations of C-peptide and proinsulin, decreased HOMA2 index, and increased QUICKI value.

Glucose-related hormone concentrations and hyperglycemia

At T1, hyperglycemic infants (N=30) had significantly higher glucose and leptin concentrations than normoglycemic infants (N=18), as well as significantly lower QUICKI value (Figure 3). At T2, hyperglycemic infants (N=22) had significantly higher C-peptide and insulin concentrations than normoglycemic infants (N=14). At both timepoints, hyperglycemic infants had significantly higher proinsulin concentrations than normoglycemic infants.

Associations between glucose-related hormone concentrations at T1 and duration of subsequent hyperglycemia

Higher glucose and proinsulin concentrations and insulin:C-peptide ratio at T1, as well as lower QUICKI value, were significantly associated with more days with hyperglycemia > 10 mmol/L during the rest of the admission period (after T1; supplementary file 2).

Confidential: For Review Only

Discussion

This prospective study reports concentrations of a broad range of glucose-related hormones in VLBW infants at two timepoints: DOL 7±3 days and PMA 36±1 weeks. Insulin resistance markers decreased while insulin sensitivity markers increased between the two timepoints. Prematurity was associated with higher concentrations of insulin resistance markers. Compared with normoglycemic infants, hyperglycemic infants had higher concentrations of a number of insulin resistance markers. Higher glucose and proinsulin concentrations and insulin:C-peptide ratio and lower QUICKI value at DOL 7 were associated with longer periods of hyperglycemia during the remaining admission period.

Glucose-related hormone concentrations reported in this study were higher than those reported in previous literature. For example, Salis et al. reported lower insulin, glucose, C-peptide and glucose concentrations, and higher GLP-1 concentrations and insulin:C-peptide ratio (28). However, substantial methodical differences (method of laboratory test, timing of blood sampling, population selection) render a comparison of such results difficult. No previous study has reported glucose-related hormone concentrations at precisely the same timepoints as the current study. Furthermore, the LIGHT-study included the largest proportion of extremely preterm infants compared with other published literature.

Concentrations of C-peptide, insulin, glucose, proinsulin, ghrelin, resistin and glucagon, as well as proinsulin:insulin ratio, have significantly decreased from DOL 7 to PMA 36 weeks, while leptin concentrations and QUICKI value significantly increased. This can be interpreted as an improvement in insulin resistance status, with a decrease in insulin resistance-promoting hormones and an increase in insulin sensitivity markers as the infant matures and the nutritional status improves. Unexpectedly, concentrations of GLP-1, an incretin hormone which is secreted in response to increased enteral nutrition and decreases insulin resistance,

Confidential: For Review Only

decreased with time. This might be due to the overall trend of decrease in insulin resistance with time which in turn would decrease the need in a GLP-1 response. These findings should be confirmed in future larger studies.

In our study, lower gestational age at birth was significantly associated with higher concentrations of glucose, ghrelin and resistin and lower concentrations of GLP-1 on DOL 7; with higher concentrations of insulin and leptin and decreased proinsulin:insulin ratio at PMA 36 weeks; and with higher concentrations of C-peptide and proinsulin, increased HOMA2 index and decreased QUICKI index at both timepoints. Overall, these results suggest an association between lower gestational age at birth and increased insulin resistance, both early in life and when nearing term age. Salis et al. have previously shown in a smaller study population that lower gestational age at birth was associated with higher insulin and glucose concentrations (25, 28).

Compared to normoglycemic infants, hyperglycemic infants had significantly higher concentrations of glucose and leptin and lower QUICKI value on DOL 7; higher concentrations of C-peptide and insulin at PMA 36 weeks; and higher concentrations of proinsulin at both timepoints. These results are supported by findings from two smaller previous studies (25, 27). This suggests that insulin resistance might be the mechanism responsible for hyperglycemia. Of note, there was no significant difference in proinsulin:insulin ratio between the groups, which does not support the theory of aberrant proinsulin processing as a cause of hyperglycemia (27). Another important finding is the relatively high HOMA2 scores noted in the different infant groups while mean glucose concentrations were not in the hyperglycemic range. This might suggest that VLBW infants have a subclinical insulin resistance even in the absence of overt hyperglycemia.

https://mc.manuscriptcentral.com/bmjpo

Confidential: For Review Only

The LIGHT-study also presents evidence for an association between glucose-related hormone concentrations and later hyperglycemia. Higher concentrations of glucose and proinsulin measured on DOL 7, as well as higher insulin:C-peptide ratio and lower QUICKI value (based on glucose and insulin concentrations) at the same timepoint, were significantly associated with higher number of days with hyperglycemia after this timepoint. This suggests that measuring these parameters at DOL 7 might help identify those infants that are most in need of close monitoring of glucose concentrations. Further studies are needed to explore the feasibility of using these markers to identify infants who have higher risk for prolonged hyperglycemia.

Only 25% of infants had quantifiable leptin concentrations on DOL 7 which increased to 92% of infants at PMA 36 weeks. Two previous studies in preterm infants have reported similar leptin concentrations in the first days of life (34, 35). Siahanido et al. on the other hand reported somewhat higher leptin concentrations at discharge (mean DOL 41) in preterm infants (36). The LIGHT-study included a higher proportion of extremely preterm infants which could explain the difference in the results, supporting in part the finding that more premature infants have increased insulin resistance and reflecting the poor nutritional status of these infants shortly after birth and its improvement during the admission period.

The LIGHT-study was a prospective study focused on glucose metabolism in VLBW infants. The many different hormonal pathways that affect glucose metabolism are intricately intertwined and this study is the first to report the results of a broad glucose-related hormone panel in this patient population. Furthermore, results are reported at two different timepoints, providing unique information regarding trends over time. To the best of our knowledge, it is the largest study to date to compare glucose-related hormone concentrations between hyperglycemic and normoglycemic infants. It is also the first study to report glucose-related

https://mc.manuscriptcentral.com/bmjpo

Confidential: For Review Only

hormone concentrations in preterm infants measured at the same predefined PMA nearing term age.

Although the largest to-date of its kind, the number of infants included in this study was relatively small, and larger studies are needed to confirm the findings. As the NICU at Umeå university hospital is one of six designated NICUs in Sweden providing care for extremely preterm infants, the LIGHT-study included a majority of extremely preterm infants (as opposed to VLBW). It should be noted that HOMA2 and QUICKI indeces, as well as the interpretation of glucose-related hormone concentrations in relation to one another in general, should usually be done using blood samples obtained in a fasting state. It would be unethical to implement this method in preterm infants and we have therefore used blood samples obtained just before enteral feedings which reflects the actual clinical situation in the NICU. Most of the infants received parenteral nutrition to some degree at T1, and two infants received parenteral nutrition at T2. This might have affected the concentrations measured to some extent. As mentioned earlier, the different hormonal pathways affecting glucose metabolism are complicated and difficult to isolate from one another. The results should therefore be interpreted with caution since it is debated whether insulin resistance markers (even in the fasting state) fully reflect the insulin resistance status in lean children.

Conclusion

VLBW infants seem to have a glucose-related hormone profile consistent with insulin resistance, both early in life and when nearing term age. Hyperglycemic preterm infants have higher concentrations of insulin resistance markers than normoglycemic infants. Lower gestational age is associated with higher concentrations of insulin resistance markers and insulin resistance decreases when nearing term age. Glucose and proinsulin concentrations,

https://mc.manuscriptcentral.com/bmjpo

Confidential: For Review Only

insulin:C-peptide ratio and QUICKI value might be useful markers to identify infants at risk for recurring hyperglycemia.

Acknowledgments: The authors would like to thank the participating parents and children, the medical staff in the neonatal units, Yonas Berhane, Carina Lagerquist, Johanna Karlsson, Ulrica Hedman, and Ida Lönnstedt for their contribution to the study, and Lilla Barnets Fond for financially supporting this study.

Funding: The study was supported by research grants from Umeå University, Region Västerbotten (RV-832421, RV-930256), Lilla Barnets Fond, Samariten Foundation (2016-0221, 2017-0275, 2018-0450), Oskar Foundation and Arnerska Research Foundation (FS 1.2.2-683-19). No involvement was made by these organizations in the study design; the collection, analysis, and interpretation of data; the writing of the report; and the decision to submit the paper for publication.

Competing interests: All authors have completed the ICMJE uniform disclosure form at http://www.icmje.org/disclosure-of-interest/ and declare: no support from any organisation for the submitted work; no financial relationships with any organisations that might have an interest in the submitted work in the previous three years; no other relationships or activities that could appear to have influenced the submitted work."

Contributors' Statement: Dr Zamir conceptualized and designed the study, collected data as well as supervised data collection, carried out the statistical analyses, drafted the initial manuscript, and reviewed and revised the manuscript. Dr Stoltz Sjöström conceptualized and designed the study, and reviewed and revised the manuscript. Dr van Den Berg acquired blood samples, and reviewed and revised the manuscript. Dr Naumburg supervised data collection and reviewed and revised the manuscript. Prof Domellöf conceptualized and designed the study, supervised data collection, and reviewed and revised the manuscript. Dr Yonas Berhane supervised data collection in Sunderbyn county hospital. Carina Lagerquist performed laboratory analyses. Ulrica Hedman coordinated study procedures at Östersund county hospital. Ida Lönnstedt and Johanna Karlsson contributed to data collection. All authors approved the final manuscript as submitted and agree to be accountable for all aspects of the work.

References

1. Pertierra-Cortada A, Ramon-Krauel M, Iriondo-Sanz M, Iglesias-Platas I. Instability of glucose values in very preterm babies at term postmenstrual age. J Pediatr. 2014;165(6):1146-53 e2.
2. Mola-Schenzle E, Staffler A, Klemme M, Pellegrini F, Molinaro G, Parhofer KG, et al. Clinically stable very low birthweight infants are at risk for recurrent tissue glucose fluctuations even after fully established enteral nutrition. Arch Dis Child Fetal Neonatal Ed. 2015;100(2):F126-31.
3. Zamir I, Tornevi A, Abrahamsson T, Ahlsson F, Engstrom E, Hallberg B, et al. Hyperglycemia in Extremely Preterm Infants-Insulin Treatment, Mortality and Nutrient Intakes. J Pediatr. 2018;200:104-10 e1.
4. Hays SP, Smith EO, Sunehag AL. Hyperglycemia is a risk factor for early death and morbidity in extremely low birth-weight infants. Pediatrics. 2006;118(5):1811-8.

Confidential: For Review Only

5. Zamir I, Stoltz Sjostrom E, Ahlsson F, Hansen-Pupp I, Serenius F, Domellof M. Neonatal hyperglycaemia is associated with worse neurodevelopmental outcomes in extremely preterm infants. Arch Dis Child Fetal Neonatal Ed. 2021;106(5):460-6.

6. Zamir I, Stoltz Sjostrom E, Edstedt Bonamy AK, Mohlkert LA, Norman M, Domellof M. Postnatal nutritional intakes and hyperglycemia as determinants of blood pressure at 6.5 years of age in children born extremely preterm. Pediatr Res. 2019;86(1):115-21.

7. Auerbach A, Eventov-Friedman S, Arad I, Peleg O, Bdolah-Abram T, Bar-Oz B, et al. Long duration of hyperglycemia in the first 96 hours of life is associated with severe intraventricular hemorrhage in preterm infants. J Pediatr. 2013;163(2):388-93.

8. Alexandrou G, Skiold B, Karlen J, Tessma MK, Norman M, Aden U, et al. Early hyperglycemia is a risk factor for death and white matter reduction in preterm infants. Pediatrics. 2010;125(3):e584-91.

9. Mohsen L, Abou-Alam M, El-Dib M, Labib M, Elsada M, Aly H. A prospective study on hyperglycemia and retinopathy of prematurity. J Perinatol. 2014;34(6):453-7.

10. Ramel SE, Long JD, Gray H, Durrwachter-Erno K, Demerath EW, Rao R. Neonatal hyperglycemia and diminished long-term growth in very low birth weight preterm infants. J Perinatol. 2013;33(11):882-6.

11. Hamayun J, Mohlkert LA, Stoltz Sjostrom E, Domellof M, Norman M, Zamir I. Association between Neonatal Intakes and Hyperglycemia, and Left Heart and Aortic Dimensions at 6.5 Years of Age in Children Born Extremely Preterm. J Clin Med. 2021;10(12).

12. Hofman PL, Regan F, Jackson WE, Jefferies C, Knight DB, Robinson EM, et al. Premature birth and later insulin resistance. N Engl J Med. 2004;351(21):2179-86.

13. Balasuriya CND, Stunes AK, Mosti MP, Schei B, Indredavik MS, Hals IK, et al. Metabolic Outcomes in Adults Born Preterm With Very Low Birthweight or Small for Gestational Age at Term: A Cohort Study. J Clin Endocrinol Metab. 2018;103(12):4437-46.

14. Bo S, Bertino E, Bagna R, Trapani A, Gambino R, Martano C, et al. Insulin resistance in pre-school very-low-birth weight pre-term children. Diabetes Metab. 2006;32(2):151-8.

15. de Jong M, Cranendonk A, van Weissenbruch MM. Components of the metabolic syndrome in early childhood in very-low-birth-weight infants and term small and appropriate for gestational age infants. Pediatr Res. 2015;78(4):457-61.

16. de Mendonca E, de Lima Macena M, Bueno NB, de Oliveira ACM, Mello CS. Premature birth, low birth weight, small for gestational age and chronic non-communicable diseases in adult life: A systematic review with meta-analysis. Early Hum Dev. 2020;149:105154.

17. Markopoulou P, Papanikolaou E, Analytis A, Zoumakis E, Siahanidou T. Preterm Birth as a Risk Factor for Metabolic Syndrome and Cardiovascular Disease in Adult Life: A Systematic Review and Meta-Analysis. J Pediatr. 2019;210:69-80 e5.

18. Mathai S, Cutfield WS, Derraik JG, Dalziel SR, Harding JE, Robinson E, et al. Insulin sensitivity and beta-cell function in adults born preterm and their children. Diabetes. 2012;61(10):2479-83.

19. Morrison KM, Ramsingh L, Gunn E, Streiner D, Van Lieshout R, Boyle M, et al. Cardiometabolic Health in Adults Born Premature With Extremely Low Birth Weight. Pediatrics. 2016;138(4).

20. Regan FM, Cutfield WS, Jefferies C, Robinson E, Hofman PL. The impact of early nutrition in premature infants on later childhood insulin sensitivity and growth. Pediatrics. 2006;118(5):1943-9.

21. Rotteveel J, van Weissenbruch MM, Twisk JW, Delemarre-Van de Waal HA. Infant and childhood growth patterns, insulin sensitivity, and blood pressure in prematurely born young adults. Pediatrics. 2008;122(2):313-21.

22. Pollak A, Cowett RM, Schwartz R, Oh W. Glucose disposal in low-birth-weight infants during steady-state hyperglycemia: effects of exogenous insulin administration. Pediatrics. 1978;61(4):546-9.

23. Blanco CL, McGill-Vargas LL, Gastaldelli A, Seidner SR, McCurnin DC, Leland MM, et al. Peripheral insulin resistance and impaired insulin signaling contribute to abnormal glucose metabolism in preterm baboons. Endocrinology. 2015;156(3):813-23.

Confidential: For Review Only

24. McGill-Vargas L, Gastaldelli A, Liang H, Anzueto Guerra D, Johnson-Pais T, Seidner S, et al. Hepatic Insulin Resistance and Altered Gluconeogenic Pathway in Premature Baboons. Endocrinology. 2017;158(5):1140-51.
25. Salis ER, Reith DM, Wheeler BJ, Broadbent RS, Medlicott NJ. Hyperglycaemic preterm neonates exhibit insulin resistance and low insulin production. BMJ Paediatr Open. 2017;1(1):e000160.
26. Bansal A, Bloomfield FH, Connor KL, Dragunow M, Thorstensen EB, Oliver MH, et al. Glucocorticoid-Induced Preterm Birth and Neonatal Hyperglycemia Alter Ovine beta-Cell Development. Endocrinology. 2015;156(10):3763-76.
27. Mitanchez-Mokhtari D, Lahlou N, Kieffer F, Magny JF, Roger M, Voyer M. Both relative insulin resistance and defective islet beta-cell processing of proinsulin are responsible for transient hyperglycemia in extremely preterm infants. Pediatrics. 2004;113(3 Pt 1):537-41.
28. Salis ER, Reith DM, Wheeler BJ, Broadbent RS, Medlicott NJ. Insulin resistance, glucagon-like peptide-1 and factors influencing glucose homeostasis in neonates. Arch Dis Child Fetal Neonatal Ed. 2017;102(2):F162-F6.
29. Lucas A, Bloom SR, Aynsley-Green A. Metabolic and endocrine events at the time of the first feed of human milk in preterm and term infants. Arch Dis Child. 1978;53(9):731-6.
30. Asplund K. Effects of postnatal feeding on the functional maturation of pancreatic islet B-cells of neonatal rats. Diabetologia. 1972;8(3):153-9.
31. Cowett RM, Oh W, Schwartz R. Persistent glucose production during glucose infusion in the neonate. J Clin Invest. 1983;71(3):467-75.
32. Sunehag A, Gustafsson J, Ewald U. Very immature infants (< or = 30 Wk) respond to glucose infusion with incomplete suppression of glucose production. Pediatr Res. 1994;36(4):550-5.
33. Ogilvy-Stuart AL, Beardsall K. Management of hyperglycaemia in the preterm infant. Arch Dis Child Fetal Neonatal Ed. 2010;95(2):F126-31.
34. Park MJ, Namgung R, Kim JN, Kim DH. Serum leptin, IGF-I and insulin levels in preterm infants receiving parenteral nutrition during the first week of life. J Pediatr Endocrinol Metab. 2001;14(4):429-33.
35. Ng PC, Lam CW, Lee CH, Wong GW, Fok TF, Wong E, et al. Changes of leptin and metabolic hormones in preterm infants: a longitudinal study in early postnatal life. Clin Endocrinol (Oxf). 2001;54(5):673-80.
36. Siahanidou T, Mandyla H, Papassotiriou GP, Papassotiriou I, Chrousos G. Circulating levels of adiponectin in preterm infants. Arch Dis Child Fetal Neonatal Ed. 2007;92(4):F286-90.

Confidential: For Review Only

Figure 1. Included and excluded very low birth weight infants. PMA – postmenstrual age; DOL – day of life.

Figure 2. Concentrations of glucose-related hormones in very low birth weight infants on day of life 7±3 (Timepoint 1; black) compared to concentrations at postmenstrual age 36±1 weeks (Timepoint 2; grey). * P<0.05; ** P<0.01; *** P<0.001. GLP-1 – glucagon-like peptide 1; HOMA2 – homeostatic model assessment 2; QUICKI – quantitative insulin sensitivity check index.

Figure 3. Concentrations of glucose-related hormones in hyperglycemic and normoglycemic very low birth weight infants sampled on day of life 7±3 (Timepoint 1; black and light grey, respectively) and at postmenstrual age 36±1 weeks (Timepoint 2; dark grey and white, respectively). * P<0.05; ** P<0.01; *** P<0.001. GLP-1 – glucagon-like peptide 1; HOMA2 – homeostatic model assessment 2; QUICKI – quantitative insulin sensitivity check index.

Confidential: For Review Only

498 **Table 1. Cohort characteristics.**

Characteristic	DOL 7 (Timepoint 1) N=48	PMA 36 weeks (Timepoint 2) N=36
**Perinatal variables**		
Gestational age, weeks, mean (SD)	27.2 (2.5)	27.3 (2.7)
Extremely preterm (< 28 weeks), N (%)	31 (65)	23 (64)
Male sex, N (%)	14 (29)	11 (31)
Multiple gestation, N (%)	6 (13)	3 (8)
Small for gestational age*, N (%)	20 (42)	16 (44)
Antenatal steroid treatment, N (%)	44 (92)	34 (94)
Amnionitis, N (%)	4 (8)	4 (11)
Preeclampsia, N (%)	11 (23)	8 (22)
Cesarean section, N (%)	36 (75)	27 (75)
Apgar 1 min, mean (SD)	5 (3)	5 (2)
Apgar 5 min, mean (SD)	7 (2)	8 (2)**
Apgar 10 min, mean (SD)	8 (2)	8 (1)
**Anthropometric variables at birth**		
Weight, grams, mean (SD)	936 (280)	925 (296)
Weight z-score, mean (SD)	-1.27 (1.52)	-1.51 (1.61)
Length, cm, mean (SD)	34.7 (3.4)	34.6 (3.7)
Length z-score, mean (SD)	-1.69 (1.91)	-1.88 (2.03)
Head circumference, cm, mean (SD)	24.4 (2.4)	24.4 (2.5)
Head circumference z-score, mean (SD)	-0.95 (0.98)	-1.05 (1.05)

Confidential: For Review Only

**Neonatal variables**		
Duration (in days) of mechanical ventilation treatment during admission period, mean (SD)	8.9 (13.6)	8.7 (13.3)
Inotrope treatment, N (%)	7 (15)	4 (11)
Treatment with systemic steroids, N (%)	11 (23)	8 (22)
Insulin treatment, N (%)	9 (19)	7 (19)
Culture-verified sepsis during admission period, N (%)	5 (10)	4 (11)
Intraventricular hemorrhage		
None, N (%)	35 (73)	26 (72)
Grade 1-2, N (%)	7 (15)	6 (17)
Grade 3-4, N (%)	6 (13)	4 (11)
Necrotizing enterocolitis, N (%)	1 (2)	0 (0)
Patent ductus arteriosus, N (%)	29 (60)	18 (50)**
Periventricular leukomalacia, N (%)	1 (2)	1 (3)
Retinopathy of prematurity		
None, N (%)	30 (63)	22 (61)
Stage 1-2, N (%)	9 (19)	6 (17)
Stage 3-5, N (%)	9 (19)	8 (22)
Bronchopulmonary dysplasia, N (%)	19 (40) (N = 47)	16 (44)

* defined as birth weight < 10th %-ile; ** P < 0.05 when comparing infants remaining in the study and excluded infants at Timepoint 2. DOL – day of life; PMA – postmenstrual age.

Confidential: For Review Only

504 **Table 2. Concentrations of glucose-related hormones in very low birth weight infants at**
505 **day of life 7±3 (Timepoint 1) and at postmenstrual age 36±1 weeks (Timepoint 2).**

	**Timepoint 1**<br>**N=48**	**Timepoint 2**<br>**N=36**
C-peptide, pmol/L, median (IQR)	440.5 (253.6-618.8)	274.7 (156.9-537.6)
Insulin, pmol/L, median (IQR)	183.8 (108.7-288.4)	80.3 (39.6-202.3)
Insulin:C-peptide, median (IQR)	0.42 (0.34-0.55)	0.35 (0.25-0.47)
Glucose, mmol/L, median (IQR)	6.5 (5.7-8.5)	5.7 (4.9-6.7)
HOMA2, median (IQR)	3.6 (2.1-5.8)	1.6 (0.8-3.8)
QUICKI, median (IQR)	0.28 (0.27-0.30)	0.32 (0.29-0.36)
Proinsulin, pmol/L, median (IQR)	42.6 (31.5-61.8)	13.4 (10.0-21.0)
Proinsulin:insulin, median (IQR)	0.27 (0.19-0.34)	0.14 (0.09-0.30)
Leptin, µg/L, median (IQR)*	0.11 (0.05-0.25)	0.55 (0.33-1.18)
Ghrelin, pmol/L, median (IQR)	1326.3 (1029.0-1694.8)	940.2 (826.9-1110.2)
GLP1, pmol/L, median (IQR)	318.3 (230.7-428.9)	261.7 (194.6-366.1)
Resistin, ng/mL, median (IQR)	29.9 (24.8-36.2)	17.9 (15.1-25.3)
Glucagon, ng/L, mean (SD)	1175.1 (264.6)	950.2 (254.0)

506 * Quantifiable concentrations of leptin were found in 12 infants (25%) at Timepoint 1 and in
507 33 infants (92%) at Timepoint 2 (Lower limit of quantification 0.01 mcg/L). IQR –
508 interquartile range; HOMA2 – homeostatic model assessment 2; QUICKI – quantitative
509 insulin sensitivity check index; GLP-1 – glucagon-like peptide.

Confidential: For Review Only

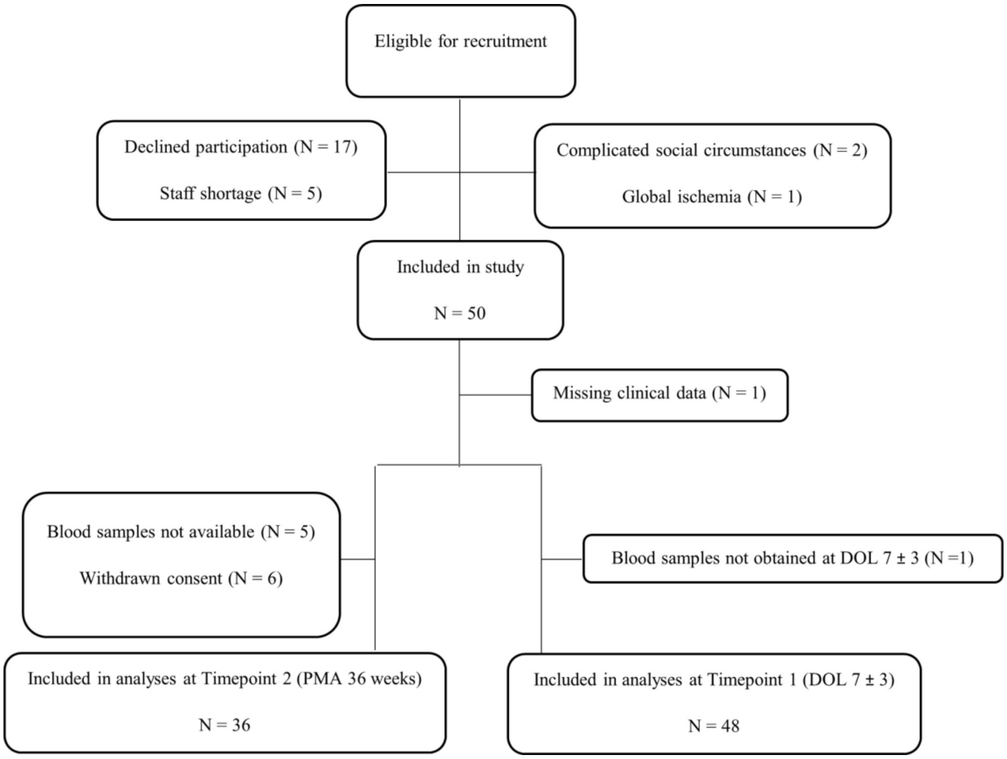

Included and excluded very low birth weight infants. PMA – postmenstrual age; DOL – day of life.

171x129mm (300 x 300 DPI)

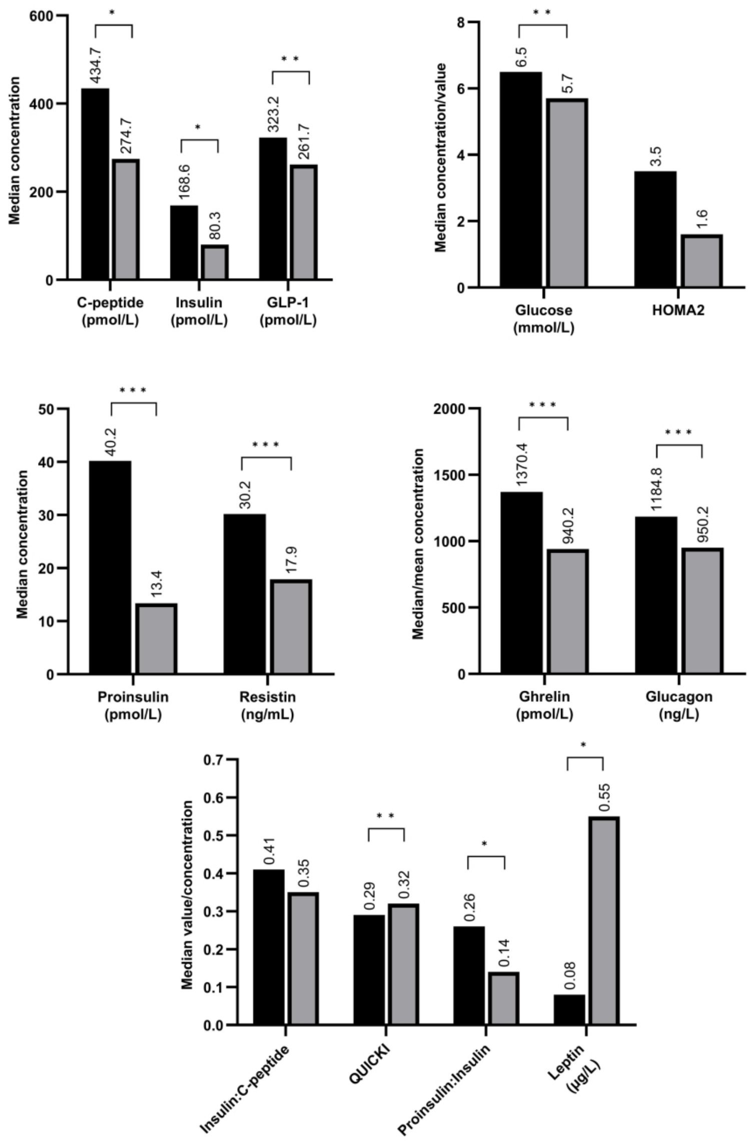

Figure 2. Concentrations of glucose-related hormones in very low birth weight infants on day of life 7±3 (Timepoint 1; black) compared to concentrations at postmenstrual age 36±1 weeks (Timepoint 2; grey). * P<0.05; ** P<0.01; *** P<0.001. GLP-1 – glucagon-like peptide 1; HOMA2 – homeostatic model assessment 2; QUICKI – quantitative insulin sensitivity check index.

190x271mm (300 x 300 DPI)

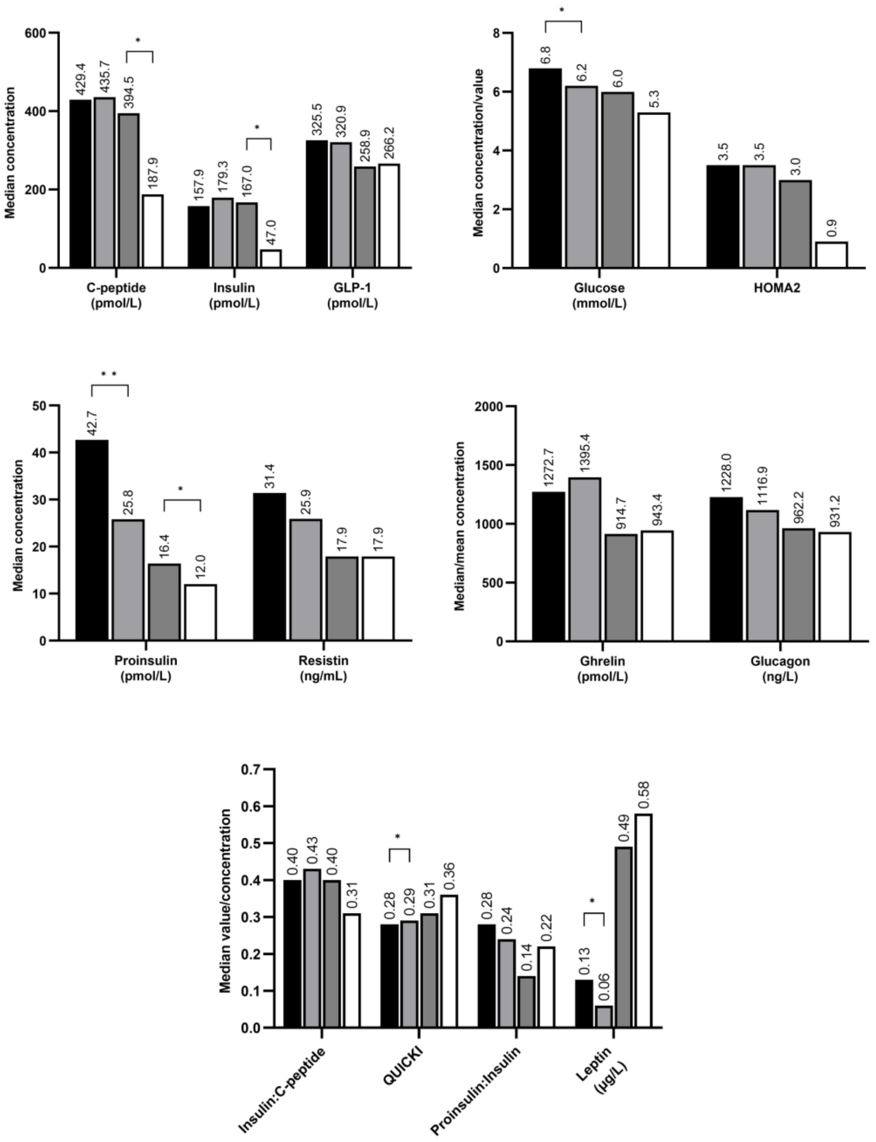

Figure 3. Concentrations of glucose-related hormones in hyperglycemic and normoglycemic very low birth weight infants sampled on day of life 7±3 (Timepoint 1; black and light grey, respectively) and at postmenstrual age 36±1 weeks (Timepoint 2; dark grey and white, respectively). * P<0.05; ** P<0.01; *** P<0.001. GLP-1 – glucagon-like peptide 1; HOMA2 – homeostatic model assessment 2; QUICKI – quantitative insulin sensitivity check index.

207x259mm (300 x 300 DPI)

Supplementary file 1. Associations between gestational age at birth and glucose-related hormone concentrations on day of life 7±3 (Timepoint 1) and at postmenstrual age 36±1 weeks (Timepoint 2) in very low birth weight infants.

	Timepoint 1			Timepoint 2		
	Coefficient	95% CI	P value	Coefficient	95% CI	P value
C-peptide	-39.7	-72.3 to -7.2	**0.02**	-51.8	-81.8 to -21.9	**0.001**
Insulin	-16.6	-34.3 to 1.2	0.07	-29.9	-49.0 to -10.7	**0.003**
Insulin: C-peptide	-0.01	-0.03 to 0.01	0.43	-0.02	-0.04 to 0.01	0.13
Glucose	-0.4	-0.7 to -0.2	**0.003**	-0.2	-0.4 to 0.01	0.07
HOMA2	-0.4	-0.7 to -0.03	**0.03**	-0.7	-1.1 to -0.3	**0.001**
QUICKI	0.006	0.002 to 0.01	**0.002**	0.012	0.005 to 0.018	**0.001**
Proinsulin	-6.1	-8.4 to -3.8	< **0.001**	-1.4	-2.5 to -0.3	**0.01**
Proinsulin: Insulin	-0.01	-0.05 to 0.02	0.44	0.02	0.00 to 0.04	**0.048**
Leptin*	-0.06	-0.29 to 0.16	0.55	-0.15	-0.2 to -0.07	**0.001**
Ghrelin	-79.0	-141.2 to -16.8	**0.01**	-1.8	-62.1 to 58.4	0.95
GLP-1	20.1	0.1 to 40.1	**0.049**	1.8	-14.5 to 18.2	0.82
Resistin	-1.6	-2.6 to -0.6	**0.002**	-0.1	-1.0 to 0.9	0.88

Confidential: For Review Only

Glucagon	-4.4	-37.7 to 29.0	0.79	12.3	-20.8 to 45.4	0.45

* Quantifiable concentrations of leptin were found in 12 infants (25%) at Timepoint 1 and in 33 infants (92%) at Timepoint 2 (LLOQ 0.01 mcg/L). HOMA2 – homeostatic model assessment 2; QUICKI – quantitative insulin sensitivity check index; GLP-1 – glucagon-like peptide.

Confidential: For Review Only

Supplementary file 2. Associations between glucose-related hormone concentrations on day of life 7±3 (Timepoint 1) and duration of subsequent hyperglycemia in very low birth weight infants.

**Glucose-related parameter (increase in value measured at Timepoint 1)**	**N days with hyperglycemia after Timepoint 1**			
	B	95% CI	P value	R^2
C-peptide (+100 pmol/L)	0.1	-0.5 to 0.7	0.68	0.004
Insulin (+100 pmol/L)	0.7	-0.3 to 1.8	0.18	0.04
Insulin:C-peptide (+0.1)	1.48	0.69 to 2.28	< **0.001**	0.24
Glucose (+1 mmol/L)	1.26	0.71 to 1.81	< **0.001**	0.32
HOMA (+1)	0.48	-0.06 to 1.02	0.08	0.07
QUICKI (+0.01)	-0.48	-0.91 to -0.05	**0.03**	0.08
Proinsulin (+10 pmol/L)	0.7	0.03 to 1.4	**0.04**	0.09
Proinsulin:insulin (+0.1)	0.03	-0.56 to 0.62	0.92	0.0
Leptin (+0.1 mcg/L)	-0.06	-0.64 to 0.51	0.82	0.001
Ghrelin (+100 pmol/L)	0.2	-0.04 to 0.5	0.09	0.06
GLP-1 (+1 pmol/L)	0.0	-0.01 to 0.01	0.95	0.0
Resistin (+1 ng/mL)	0.11	-0.08 to 0.29	0.25	0.03
Glucagon (+100 ng/L)	0.1	-0.5 to 0.7	0.84	0.001

HOMA2 – homeostatic model assessment 2; QUICKI – quantitative insulin sensitivity check index; GLP-1 – glucagon-like peptide.

Confidential: For Review Only
