# Supplementary material for: Insulin resistance prior to term age in very low birthweight infants: a prospective study
Source: BMJ Paediatr Open. 2024 Feb 10;8(1):e002470. doi: 10.1136/bmjpo-2023-002470 (PMC10862284; doi:10.1136/bmjpo-2023-002470)
Supplement: Reviewer comments [file bmjpo-2023-002470.reviewer_comments.pdf]

PEER REVIEW HISTORY

BMJ Paediatrics Open publishes all reviews undertaken for accepted manuscripts. Reviewers are asked to complete a checklist review form and are provided with free text boxes to elaborate on their assessment. These free text comments are reproduced below.

ARTICLE DETAILS

**TITLE (PROVISIONAL)**	Insulin resistance prior to term age in very low birth infants: a prospective study
**AUTHORS**	Zamir, Itay<br>Stoltz Sjöström, Elisabeth<br>van Den Berg, Johannes<br>Naumburg, Estelle<br>Domellof, Magnus

This article was not externally reviewed at BMJ Paediatrics Open. Protocols that have been independently assessed prior to submission to BMJ Paediatrics Open are usually fast-tracked to publication on the grounds that further substantial changes will not be possible. This independent assessment will usually be external, independent review for both a substantial grant award from a non-commercial or government funder and ethics approval.
